# Cannabinoid receptor 1 signalling modulates stress susceptibility and microglial responses to chronic social defeat stress

**DOI:** 10.1038/s41398-021-01283-0

**Published:** 2021-03-15

**Authors:** Eva C. Beins, Thomas Beiert, Imke Jenniches, Jan N. Hansen, Este Leidmaa, Jan W. Schrickel, Andreas Zimmer

**Affiliations:** 1 Institute of Molecular Psychiatry, Medical Faculty, University of Bonn, Bonn, Germany; 2 Institute of Human Genetics, Medical Faculty, University of Bonn, Bonn, Germany; 3 Medizinische Klinik und Poliklinik II, University Hospital Bonn, Bonn, Germany; 4 Institute of Innate Immunity, Medical Faculty, University of Bonn, Bonn, Germany

**Keywords:** Physiology, Molecular neuroscience

## Abstract

Psychosocial stress is one of the main environmental factors contributing to the development of psychiatric disorders. In humans and rodents, chronic stress is associated with elevated inflammatory responses, indicated by increased numbers of circulating myeloid cells and activation of microglia, the brain-resident immune cells. The endocannabinoid system (ECS) regulates neuronal and endocrine stress responses via the cannabinoid receptor 1 (CB1). CB1-deficient mice (*Cnr1*^−/−^) are highly sensitive to stress, but if this involves altered inflammatory responses is not known. To test this, we exposed *Cnr1*^+/+^ and *Cnr1*^−/−^ mice to chronic social defeat stress (CSDS). *Cnr1*^−/−^ mice were extremely sensitive to a standard protocol of CSDS, indicated by an increased mortality rate. Therefore, a mild CSDS protocol was established, which still induced a behavioural phenotype in susceptible *Cnr1*^−/−^ mice. These mice also showed altered glucocorticoid levels after mild CSDS, suggesting dysregulation of the hypothalamic–pituitary–adrenal (HPA) axis. Mild CSDS induced weak myelopoiesis in the periphery, but no recruitment of myeloid cells to the brain. In contrast, mild CSDS altered microglial activation marker expression and morphology in *Cnr1*^−/−^ mice. These microglial changes correlated with the severity of the behavioural phenotype. Furthermore, microglia of *Cnr1*^−/−^ mice showed increased expression of *Fkbp5*, an important regulator of glucocorticoid signalling. Overall, the results confirm that CB1 signalling protects the organism from the physical and emotional harm of social stress and implicate endocannabinoid-mediated modulation of microglia in the development of stress-related pathologies.

## Introduction

Stress is defined as a state in which homoeostasis is threatened by external or internal potentially harmful stimuli^1^. The body’s response to these stressors includes the activation of the hypothalamic–pituitary–adrenal (HPA) axis and the sympathetic nervous system (SNS), which both influence behavioural and immunological responses to stress. If homoeostasis is not restored after cessation of the stressor or disrupted over a long period, these responses (e.g. elevated glucocorticoid (GC) levels) become harmful and can result in disease. Thus, chronic stress is one of the major environmental factors in the development of affective disorders, such as anxiety and depression^2^. However, the underlying mechanisms are still not fully understood. In the last decades, the importance of inflammatory processes in these disorders has become increasingly evident^3,4^. While GCs are generally potent immunosuppressants, chronic stress is associated with GC resistance and exacerbated inflammatory processes, such as increased myeloid cell counts and elevated levels of circulating interleukin-6 (IL-6)^5,6^.

Rodent models of chronic social stress recapitulate many of the behavioural, endocrine, and immunological symptoms associated with chronic stress and neuropsychiatric disorders in humans. For example, repeated social defeat stress induces the production of myeloid cells in the bone marrow and trafficking of inflammatory monocytes to other organs, including the brain^7,8^. The proliferation of hematopoietic stem cells and the mobilisation of monocytes from the bone marrow during chronic stress is dependent on adrenergic^6^ as well as GC signalling^9^. In the brain, chronic stress induces neuroinflammation and activates microglia—the brain-resident immune cells^10–14^. The functional relevance of microglia in depression-related pathologies was further repeatedly demonstrated in other studies using genetic and pharmacological inhibition of microglia activation^15–17^.

The endocannabinoid system (ECS), a neuromodulatory signalling system, is activated after stress exposure and has homoeostatic functions^18–20^. The ECS comprises the cannabinoid receptors CB1 and CB2, which are activated by the endocannabinoids 2-arachidonoylglycerol (2-AG) and N-arachidonoyl ethanolamine (anandamide; AEA). The CB1 receptor is abundantly expressed in the central and peripheral nervous system, where it modulates synaptic transmission by suppressing excessive neurotransmitter release via retrograde negative feedback^21^. Its expression is especially high in brain regions associated with the regulation of emotion, mood, cognition and stress responses, suggesting a prominent role of CB1 signalling in modulating these processes^22^. In line with this, pharmacological blockade or genetic disruption of CB1 is associated with increased anxiety-, and depressive-like behaviour, impaired extinction of aversive memories and increased stress sensitivity^22,23^. CB1 signalling is essentially involved in regulating stress responses by modulating the fast feedback inhibition of the HPA axis and its adaptation during repeated stress exposure^24–30^. Furthermore, CB1 receptors are expressed on adrenergic and noradrenergic neurons both in the brain and peripheral nerves, where their activation inhibits noradrenaline (norepinephrine, NE) release^31–36^. Although it is known that CB1 signalling regulates neuronal and endocrine stress responses, it is largely unknown if and how it affects immunological responses to stress and whether these contribute to the increased stress-susceptibility in the absence of CB1 signalling. Generally, blocking CB1 signalling in peripheral organs appears to be protective under chronic inflammatory conditions, such as liver fibrosis, where the protective effects are mediated by reducing macrophage migration and pro-inflammatory activation^37,38^. In the brain, lack of CB1 receptors on GABAergic forebrain neurons alters neuroinflammatory processes and microglial activation at baseline as well as during ageing and in response to pro-inflammatory stimuli^39,40^. CB1 activation was further shown to protect against immobilisation/acoustic stress-induced excitotoxicity and neuroinflammation in the prefrontal cortex (PFC)^41^. Additionally, it was recently shown that cannabinoid agonist treatment during repeated social defeat can attenuate stress-induced neuroinflammation and anxiety-like behaviour^42^. Whether these effects were mediated by CB1 or CB2 receptors is, however, not known.

In this study, we analysed the behavioural, endocrine, and inflammatory responses to chronic social defeat stress (CSDS) in CB1-deficient mice (*Cnr1*^−/−^) and their wild type littermates (*Cnr1*^+/+^). We show that *Cnr1*^−/−^ mice are highly susceptible to CSDS, which is accompanied by altered GC signalling, minor changes in peripheral myeloid cell populations and altered microglial function.

## Materials and methods

### Mice

Eight- to ten-week-old male *Cnr1*^−/−^ and *Cnr1*^+/+^ littermates were used for the experiments (strain B6.cg Cnr1 tm1Zim^43^). Male CD1 aggressor mice (retired breeders) were obtained from a commercial breeder (Janvier). Mice were housed in standard laboratory cages under specific pathogen free conditions with *ad libitum* access to food and water. At least 1 week before starting the experiments, mice were habituated to a reversed light-dark cycle (lights on at 9 p.m., lights off at 9 a.m.). All experiments followed the guidelines of the German Animal Protection Law and were approved by the Local Committee for Animal Health, LANUV NRW (84-02.04.2015.A192). Detailed information on the mouse lines and cohorts are provided in the supplementary materials.

### Chronic social defeat stress (CSDS)

In the standard CSDS paradigm, mice were subjected to daily sessions of 5–10 min of social defeat by a novel CD1 aggressor on 10 consecutive days, as described elsewhere^44^. A mild CSDS paradigm was established later, with only 1–2 min of defeat per day. Defeat sessions were performed at the beginning of the active (dark) phase. After each session, animals were separated by a transparent perforated plastic wall to allow sensory, but not physical contact for the following 24 h. Control mice were housed with two unfamiliar mice per cage, separated by a perforated wall. Subsequent to the last defeat session, mice were single-housed and tested in different stress-related behavioural paradigms (open field, social avoidance, sucrose preference, nestlet test), as described in the supplementary materials.

### Stress scoring system

To determine the overall effects of stress and genotype on behaviour, a stress score was calculated for each mouse from all behavioural test results. For each parameter, a score from zero to three was assigned, based on the deviation from wild type control values (0 = within 1x standard deviation (s.d.), 1 = more than 1x s.d., 2 = more than 2x s.d., 3 = more than 3x s.d). The stress score was calculated as the mean of all individual scores.

### Telemetric measurement of heart activity

To analyse cardiac activity of mice during CSDS, long-term electrocardiograms (ECG) were recorded in *Cnr1*^−/−^ mice (*n* = 7). Two weeks before starting the CSDS paradigm, mice were implanted with telemetric ECG-transmitters specifically made for small rodents (Data Science International, St. Paul, USA). A detailed description of the surgical procedure is provided in the supplementary materials.

### Tissue collection and isolation of cells

One day after the last defeat, mice were deeply anaesthetised and perfused with ice-cold PBS. Blood, bone marrow, spleen and brain tissues were collected and processed for flow cytometry, gene expression analysis (qPCR) or immunohistochemistry, as described in the supplementary materials.

### Flow cytometry

Cells were incubated with Fc-block (anti-mouse CD16/32) followed by primary antibody staining. Antibodies used were anti-mouse CD45, CD11b, Ly6C, CCR2, CD11c, CD115, MerTK, MHCII, lineage (CD3, CD19, NK1.1, Ly6G, TER-119), followed by APC/Cy7 streptavidin incubation for detection of biotinylated lineage antibodies (see Supplementary Table 1 for antibody details). Erythrocytes from blood, bone marrow and spleen tissue were lysed in 1x RBC-lysis buffer. Dead cells were stained using DRAQ7^TM^. Flow cytometric analysis was performed using a BD FACSCanto II and FACSDiva Software (BD). Samples with less than 5000 total live cells were excluded from analysis. Resulting files were analysed using FlowJo version 10.0.6 (Tree Star Inc.).

### RNA-isolation, reverse transcription and quantitative real-time PCR (qPCR)

Total RNA from cells or frozen tissue was extracted using Trizol^®^ Reagent (Life Technologies) according to the manufacturer’s instructions. RNA was digested with recombinant DNase I (Sigma-Aldrich) and reverse transcribed into cDNA using SuperScript™ II Reverse Transcriptase (Invitrogen). Expression of mRNA was analysed using TaqMan^®^ Gene Expression Master Mix and Gene Expression Assays (Applied Biosystems™). qPCR was performed using the LightCycler 480 (Roche). Assays used were *Cxcl12* (Mm00445553_m1) and *Hprt1* (Mm03024075_m1) as reference gene. Data were normalised to *Cnr1*^+/+^ unstressed controls.

### IL-6 ELISA

Plasma IL-6 was measured using the Mouse IL-6 ELISA Ready-SET-Go!^®^ Kit (eBioscience), following the manufacturer’s instructions. Samples and standards (4–500 pg/ml) were diluted in assay diluent and tested in duplicates.

### Corticosterone ELISA

Plasma was prepared from blood collected by cardiac puncture ~24 h after the last defeat session (9:00–10:00 a.m.). Faeces were collected during the 24 h period of single-housing after the last defeat session. Plasma and faecal corticosterone levels were measured using the DetectX^®^ Corticosterone Enzyme Immunoassay Kit (Arbour Assays), according to the manufacturer’s protocol. Steroids were extracted from dried faeces using 100% EtOH, concentrated by vacuum evaporation, dissolved in 100% EtOH and diluted with assay buffer. Plasma samples were incubated with dissociation reagent and diluted with assay buffer. Samples and standards (78.128–10000 pg/ml) were tested in duplicates.

### Immunohistochemistry, image acquisition and analysis

For analysis of microglia and neurovascular changes, free-floating 60 µm brain sections were stained for IBA1, ICAM-1 and CD45 and imaged using a Leica TCS SP8 confocal microscope. For overviews, images were acquired with a 20x objective. For microglia morphology analysis, z-stacks of 40–50 µm were acquired using the 63x objective. Images were analysed using Fiji (ImageJ 2.0.0). 3D reconstruction and analysis of microglia was performed using custom-written ImageJ plug-ins, as described previously^45^. To analyse SNS innervation of the bone marrow, longitudinal 10 µm cryosections of post-fixed, decalcified tibias were stained for tyrosine hydroxylase (TH) positive nerve endings. Z-stacks of 10 µm were acquired using the 63x objective. Next to fluorescent TH and DAPI, a transmitted light picture was taken for identification of blood vessels. A detailed description of the procedures and imaging settings is provided in the supplementary materials.

### Isolation of microglia for RNA-sequencing

Microglia (CD11b + cells) were isolated from brain tissue of stress-naïve male *Cnr1*^*+/+*^ and *Cnr1*^−/−^ mice (*n* = 3 per genotype) by magnetic cell separation. Briefly, mice were deeply anaesthetised and perfused with ice-cold PBS. Brains were collected in FACS buffer and mechanically dissociated in glass douncers in 5 ml FACS buffer + 20 µg/ml DNase I (Roche). Homogenates were filtered through a 70 µm cell strainer, washed and centrifuged (300 × *g*, 10 min, 4 °C). For myelin removal, cells were resuspended in 40% Percoll and centrifuged (300 × *g*, 30 min, 4 °C), after which myelin and supernatant were removed and pelleted cells washed in FACS buffer. CD11b + cells were isolated using CD11b MicroBeads (MACS^®^, Miltenyi Biotec) according to the manufacturer’s instructions. Isolated cell pellets were snap-frozen in liquid nitrogen and stored at −80 °C.

### RNA-sequencing and analysis

Total RNA was isolated from isolated microglia using the Qiagen RNeasy Mini Kit according to the manufacturer’s instructions and eluted in 30 µl of RNAse-free water. Subsequent processing of RNA was performed at the NGS core facility of the University of Bonn. Quality control was performed using an Agilent TapeStation. Libraries for sequencing were prepared using the QuantSeq 3′ mRNA-Seq Library Prep Kit FWD for Illumina (Lexogen) as per manufacturer instructions. Reads were sequenced on an Illumina HiSeq 2500 V4 in high output mode. Approximately 10 million single-reads of 50 bp length were obtained per sample library. RNA-Seq data can be accessed under GSE152266. Data analysis was performed using the Lexogen QuantSeq pipeline implemented in Partek^®^ Flow^®^ Genomic Analysis Software, with small modifications. A description of the analysis is provided in the supplementary materials.

### Statistical analysis

Statistical analysis was performed using Prism 6 (GraphPad). Statistical outliers were identified using the ROUT method (Q = 1%). Normality testing was performed using D’Agostino & Pearson for group sizes *n* > 7 and Shapiro–Wilk test for *n* < 7. Data were mostly analysed using two-way ANOVA, followed by Bonferroni multiple comparisons test. If more than one group did not follow normal distribution, Kruskal–Wallis test with Dunn’s multiple comparisons test was used. Correlation with behavioural stress scores was performed using Spearman correlation. *P* values < 0.05 were considered statistically significant. Statistical tests and results are reported in the figure legends.

## Results

### CB1-deficiency increases mortality rates upon severe stress exposure

**Fig. 1:**
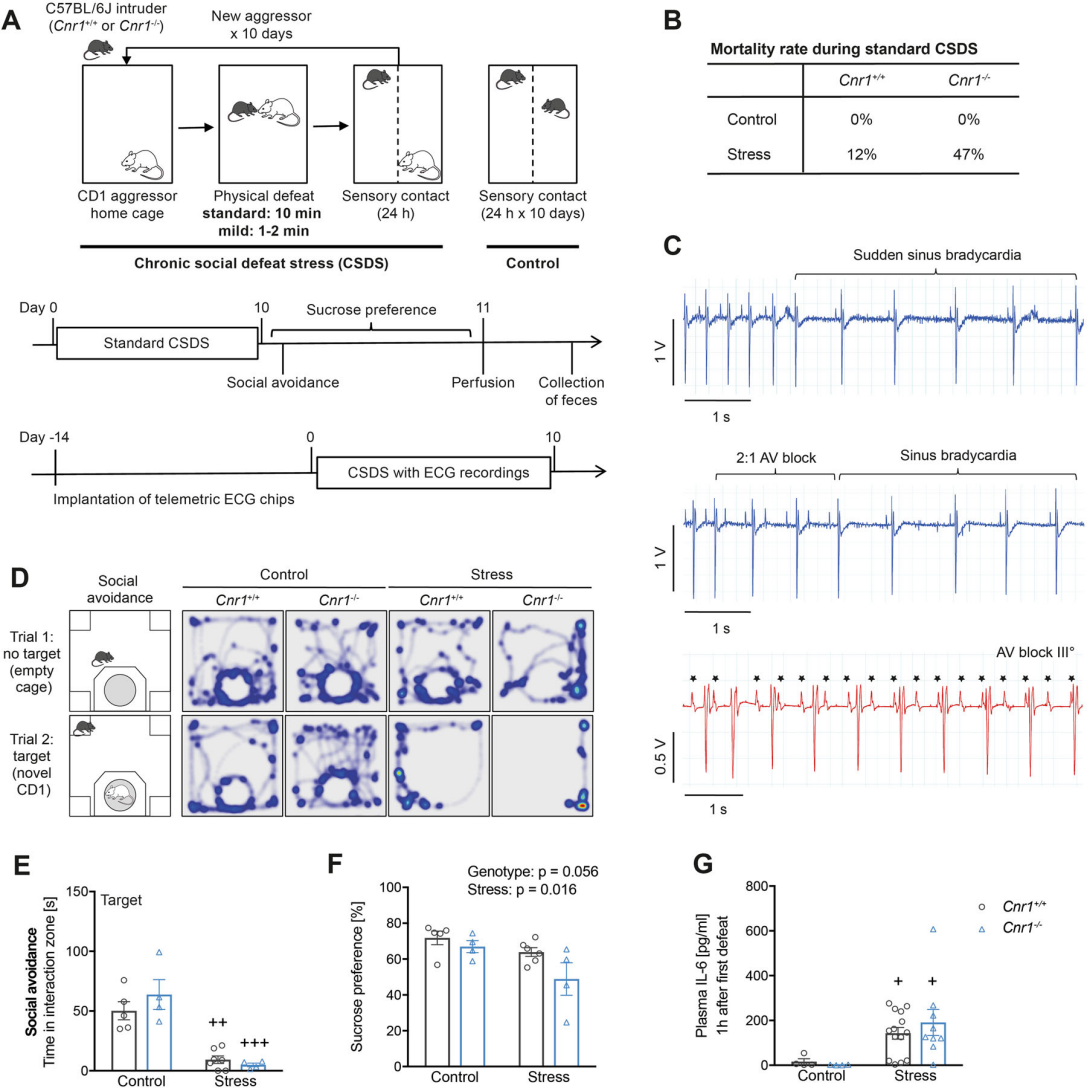
*Cnr1*^−/−^ mice show increased mortality rates during standard CSDS. **A** Experimental setup for standard and mild CSDS. **B** Mortality rates of mice during standard CSDS. **C** Exemplary traces of ECG recordings of two *Cnr1*^−/−^ mice that died during standard CSDS exposure. Top panel: example of a mouse with sudden development of sinus bradycardia. Middle panel: initial sinus rhythm (beat 1–2) with development of a short period of 2:1 atrioventricular (AV) block (beat 3–5), devolving into sinus bradycardia. Lower panel: development of a total AV block (third degree, III°). Note the complete dissociation of atrial (P wave, indicated by stars) and ventricular (QRS complex) signals. **D** Representative heatmap visualisation of the social avoidance test, illustrating the position of mice during the two trials of the test. **E** Social avoidance behaviour, measured as interaction time with a target CD1 mouse in surviving mice after standard CSDS (stress: *F*_(1,16)_ = 54.43, *p* < 0.0001). **F** Anhedonia was measured in the sucrose preference test (stress: F_(1,15)_ = 7.31, *p* = 0.016; genotype: F_(1,15)_ = 4.31, *p* = 0.056). **G** Plasma IL-6 was measured 1 h after the first 10 min stress exposure (stress: F_(1,26)_ = 14.98, *p* = 0.0007; the outlier in the *Cnr1*^−/−^ stress group (607 pg/ml) was not included during statistical analysis). Data were analysed by two-way ANOVA followed by Bonferroni post-hoc test for multiple comparison. Post-hoc significances shown in graph: *p < 0.05 vs*. Cnr1*^+/+^ of the same condition; +*p* < 0.05 vs. control of the same genotype.

In the first set of experiments, *Cnr1*^+/+^ and *Cnr1*^−/−^ mice were submitted to a standard protocol of CSDS, with 5–10 min of daily defeat sessions (Fig. 1A). However, nearly 50% of *Cnr1*^−/−^ died during the first days of stress exposure (Fig. 1B), whereas the mortality rate for *Cnr1*^+/+^ mice was ~10%. A similar increase in mortality rates was also observed during standard CSDS in mice that lack DAGLα, the main 2-AG synthesising enzyme (*Dagla*^−/−^ 30%, *Dagla*^fl/fl^ controls 7%, Supplementary Fig. 1). The animals did not die during the physical defeat, but rather during the subsequent 24-hour period of sensory contact. Since social defeat models are associated with high incidence of cardiac arrhythmias^46^, heart activity was analysed in *Cnr1*^−/−^ mice during CSDS using long-term telemetric ECG recordings (Fig. 1C). All mice that died during the experiment were bradycardic on the day of death and showed a third degree atrioventricular (AV) block, indicated by a complete dissociation of atrial and ventricular signals.

Behavioural analysis of surviving mice showed clear stress effects in the social avoidance test, indicated by significantly reduced interaction with a target CD1 mouse and increased time spent in corners for both genotypes (Fig. 1D, E, Supplementary Fig. 2). A significant main effect of stress was also detected for sucrose preference (Fig. 1F). Overall, there were no clear differences in the stress-induced behaviour between *Cnr1*^−/−^ and *Cnr1*^+/+^ mice after standard CSDS, which may have been due to a ceiling effect and the loss of highly susceptible *Cnr1*^−/−^ mice. Additional data on behavioural, endocrine, and immunological effects of standard CSDS are provided in Supplementary Fig. 2. Previous studies have shown that high susceptibility to CSDS is correlated with increased IL-6 production by peripheral leucocytes^47^. Furthermore, high levels of IL-6 are associated with increased risk of cardiovascular mortality in humans^48^. Therefore, blood was collected from submandibular veins 1 h after the first stress exposure to determine plasma concentrations of IL-6. Analysis revealed a main effect of stress, with stressed mice of both genotypes showing significantly higher IL-6 levels than unstressed controls (Fig. 1G). In our study, plasma levels of IL-6 were however not correlated to behavioural stress-susceptibility (Supplementary Fig. 3). Interestingly, one *Cnr1*^−/−^ mouse that was found dead on the following day showed extremely high plasma IL-6 levels (607 pg/ml) (Fig. 1G).

### Cnr1^−/−^ mice are more susceptible to mild CSDS

**Fig. 2:**
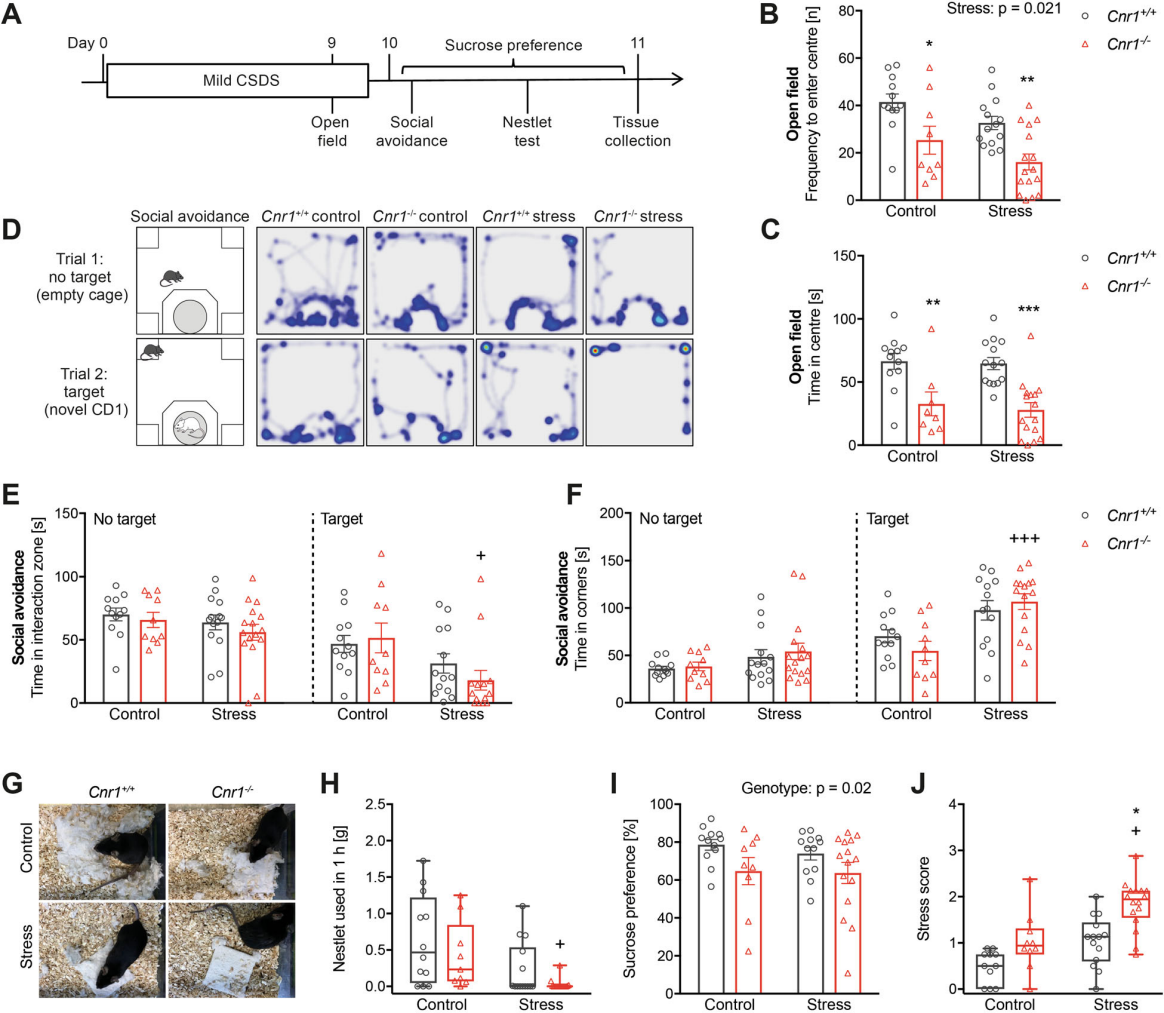
*Cnr1*^−/−^ mice are behaviourally more susceptible to mild CSDS. **A** Timeline of the mild CSDS paradigm and subsequent behavioural experiments. See Fig. 1A for detailed experimental setup. **B**, **C** Anxiety-like behaviour in the open field test, was evaluated by the frequency to enter the centre (genotype: *F*_(1,47)_ = 18.63, *p* < 0.0001; stress: *F*_(1,47)_ = 5.67, *p* < 0.021) and the time spent in the centre (genotype: *F*_(1,46)_ = 29.96, *p* < 0.0001). **D** Representative heatmap visualisation of the social avoidance test, illustrating the position of the mice during the two trials of the test. **E** Interaction with an empty cage (no target; no significant main effects) or interaction with a novel CD1 mouse (target; stress: *F*_(1,45)_ = 8.50, *p* = 0.006). **F** Time spent in corner zones with no target (no significant main effects) or with a CD1 target present (stress: F_(1,46)_ = 19.23, *p* < 0.0001). **G**, **H** Nest building behaviour was measured after mild CSDS (Kruskal–Wallis statistic = 14.43, *p* = 0.006). **I** Anhedonia was analysed using the sucrose preference test (genotype: *F*_(1,44)_ = 5.82, *p* = 0.020). **J** Behavioural stress scores were calculated for individual mice based on their performance in all behavioural tests, ranging from zero (no phenotype) to three (strong phenotype) (Kruskal–Wallis statistic = 25.1, *p* < 0.0001). Data were analysed by two-way ANOVA followed by Bonferroni post-hoc test for multiple comparison or by non-parametric Kruskal–Wallis test followed by Dunn’s multiple comparisons. Post-hoc significances shown in graph: *p < 0.05 vs*. Cnr1*^+/+^ of the same condition; +*p* < 0.05 vs. control of the same genotype.

In order to prevent mortality of *Cnr1*^−/−^ mice, we adjusted the CSDS paradigm to a much milder version by limiting the daily defeat sessions to 1–2 min (Figs. 1A and 2A). In the social avoidance test, mild CSDS had no effect on exploratory activity (empty cage–no target), but *Cnr1*^−/−^ mice showed significantly reduced interactions with a CD1 target (Fig. 2E) and increased time spent in corners (Fig. 2F). In contrast, the differences were not significant for *Cnr1*^+/+^ mice. In the open field test, significant stress and genotype effects were detected for the frequency to enter the centre of the arena (Fig. 2B). Further, control and stressed *Cnr1*^−/−^ mice spent significantly less time in the centre than corresponding *Cnr1*^+/+^ mice, indicating increased baseline anxiety-like behaviour (Fig. 2C). Nest building behaviour, a sensitive readout for home cage activity and self-care, was also reduced after stress exposure and importantly, the majority of stressed *Cnr1*^−/−^ mice did not start building a nest at all (Fig. 2G, H). Non-parametric analysis revealed a significant reduction of nest building between control and stressed *Cnr1*^−/−^ mice. Mild CSDS had no effect on sucrose preference, but *Cnr1*^−/−^ mice generally showed a reduced sucrose preference (Fig. 2I). We observed that these other behavioural domains were not correlated with stress-susceptibility in the social avoidance test, which is typically used to discriminate between stress susceptible and resilient mice (Supplementary Fig. 3). To have an overall readout of behavioural stress-susceptibility, we therefore developed a behavioural stress scoring system that incorporates the performance in all behavioural tests (Fig. 2J). Here, a significant stress effect was only observed in *Cnr1*^−/−^ mice.

### Cnr1^−/−^ mice show altered HPA axis activity after mild CSDS

**Fig. 3:**
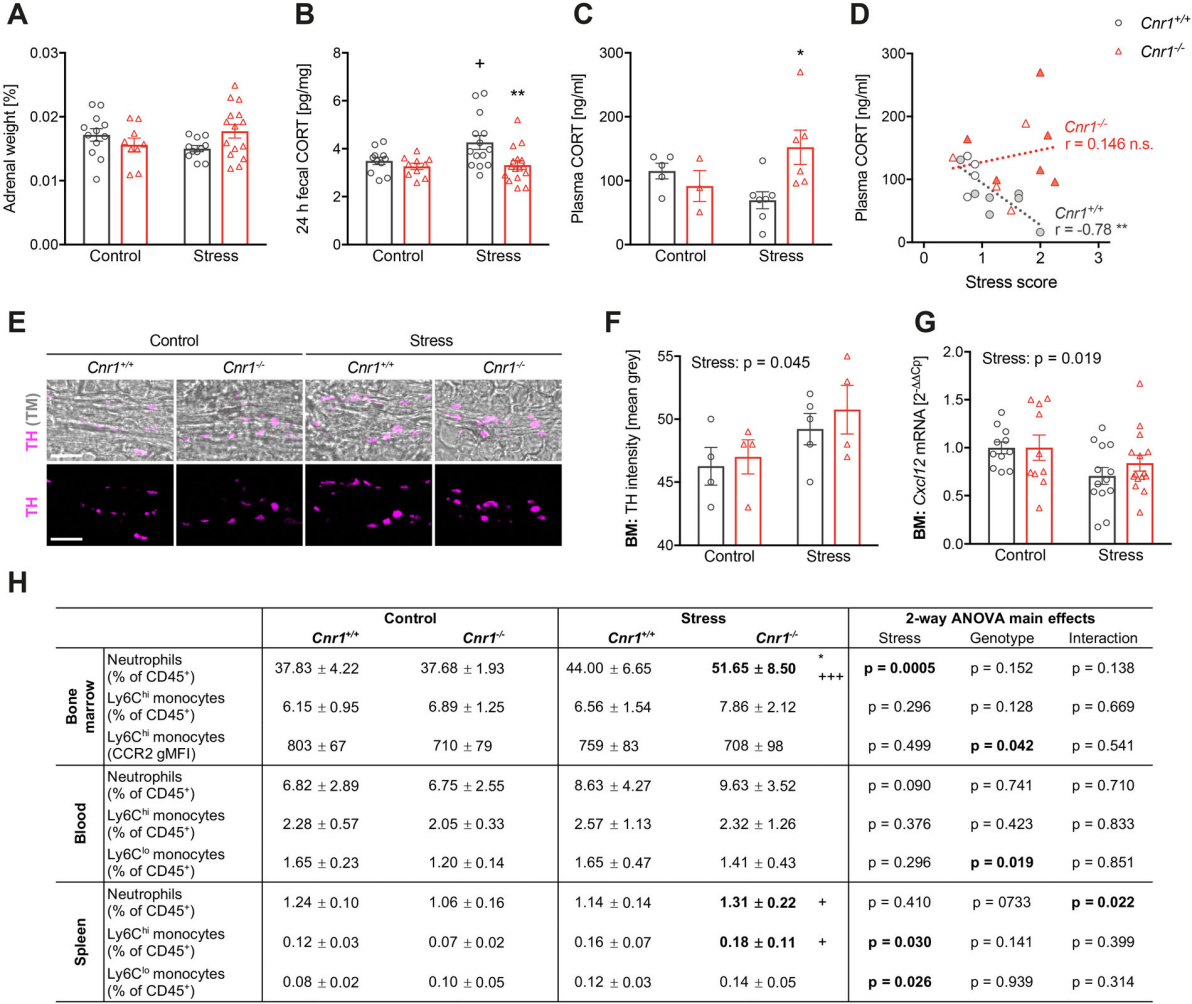
Peripheral effects of mild CSDS. **A** Adrenal glands were harvested and weighed after mild CSDS (stress x genotype interaction: *F*_(1,43)_ = 4.74, *p* = 0.035). **B** Cumulative faecal CORT excretion was measured in faeces collected from a 24 h period after mild CSDS (stress: *F*_(1,47)_ = 3.56, *p* = 0.065; genotype: *F*_(1,47)_ = 7.91, *p* = 0.010). **C** Circulating corticosterone (CORT) was measured in plasma collected ~24 h after the last stress exposure, at the beginning of the active (dark) phase (stress x genotype interaction: *F*_(1,17)_ = 6.41, *p* = 0.022). **D** Plasma CORT levels were correlated to the behavioural stress scores. Spearman correlation was performed for each genotype, independent from stress (*Cnr1*^+/+^: *r* = −0.78, *p* = 0.004; *Cnr1*^−/−^: *r* = 0.146, *p* = 0.686). **E**, **F** Tyrosine hydroxylase (TH) was stained to identify noradrenergic nerve terminals innervating the bone marrow. **E** Representative images showing TH-positive nerve endings surrounding blood vessels within the bone marrow. Upper panels show transmitted light (TM) image and TH signals (magenta); bottom: only TH signal for better visualisation; scale bar = 5 µm. **F** TH + nerve endings were identified by applying an appropriate threshold and TH immunoreactivity (mean grey) was quantified within TH + nerve endings (stress: *F*_(1,13)_ = 4.942, *p* = 0.0446). **G** *Cxcl12* mRNA expression was analysed in isolated bone marrow (BM) using qPCR, data were normalised to the expression of reference gene *Hprt1* (stress: *F*_(1,45)_ = 5.97, *p* = 0.019). **H** Leucocytes from BM, blood and spleen were isolated ~24 h after the last stress exposure and analysed by flow cytometry. Lineage stain included antibodies against CD3, CD19, NK1.1, TER-119, Ly6G and DRAQ7™ for staining dead cells. Neutrophils were identified as CD11b^+^, Lineage/Ly6G^+^, Ly6C^int^. Monocytes were identified as CD11b^+^, Lineage/Ly6G^neg^, CD115^+^ and divided into Ly6C^hi^ and Ly6C^lo^ subsets. Significant differences are indicated by bold font. Data were analysed by two-way ANOVA followed by Bonferroni post-hoc test for multiple comparison. Post-hoc significances shown in graph: *p < 0.05 vs*. Cnr1*^+/+^ of the same condition; +*p* < 0.05 vs. control of the same genotype.

We next analysed activity of the HPA axis, the major neuroendocrine stress response system. In the present study, only standard CSDS caused adrenal hypertrophy (Supplementary Fig. 2), whereas mild CSDS had no effect on adrenal gland size (Fig. 3A). Analysis of corticosterone (CORT) in faecal samples collected during the 24-hour period after the last mild CSDS exposure revealed a stress-induced increase in cumulative CORT excretion in *Cnr1*^+/+^, but not in *Cnr1*^−/−^ mice (Fig. 3B). A similar result was observed after standard CSDS (Supplementary Fig. 2). Circulating CORT levels were measured ~24 h after the last mild CSDS exposure at beginning of the active phase. At this time point, plasma CORT was significantly higher in stressed *Cnr1*^−/−^ mice compared to stressed *Cnr1*^+/+^ mice (Fig. 3C). In *Cnr1*^+/+^ mice, plasma CORT was further negatively correlated with the behavioural stress scores, i.e. most susceptible mice showed the lowest CORT levels (Fig. 3D). In contrast, there was no correlation in *Cnr1*^−/−^ mice (Fig. 3D) and no correlation with faecal CORT levels (data not shown).

### Mild CSDS induces moderate myelopoiesis in the periphery

During chronic stress, both CORT and NE can induce proliferation and egress of myeloid cells/monocytes from the bone marrow via downregulation of the retention factor CXCL12^6,9^. We therefore stained bone marrow sections for TH, the rate-limiting enzyme in NE synthesis. After mild CSDS, TH immunoreactivity was slightly increased in TH-positive nerve endings innervating the bone marrow, suggesting enhanced NE production (Fig. 3H, I). Gene expression of *Cxcl12* in the bone marrow was reduced after mild CSDS (Fig. 3J), with no differences between the genotypes. We next analysed myeloid cell populations in the bone marrow, blood and spleen using flow cytometry. Overall, effects of mild CSDS on peripheral myeloid cells were relatively small compared to previous reports in the literature and changes observed after standard CSDS (Supplementary Fig. 2). After mild CSDS, we observed significantly increased bone marrow neutrophil frequencies in *Cnr1*^−/−^ mice (Fig. 3K). In contrast, stress had no effect on Ly6C^hi^ monocyte numbers in the bone marrow or in circulation, but significantly increased splenic Ly6C^hi^ monocytes in *Cnr1*^−/−^ mice. Independent from stress, we found that *Cnr1*^−/−^ mice had reduced CCR2 expression on Ly6C^hi^ monocytes in the bone marrow and less circulating Ly6C^lo^ patrolling monocytes.

### Mild CSDS does not induce infiltration of myeloid cell to the brain, but alters microglia activation in Cnr1^−/−^ mice

**Fig. 4:**
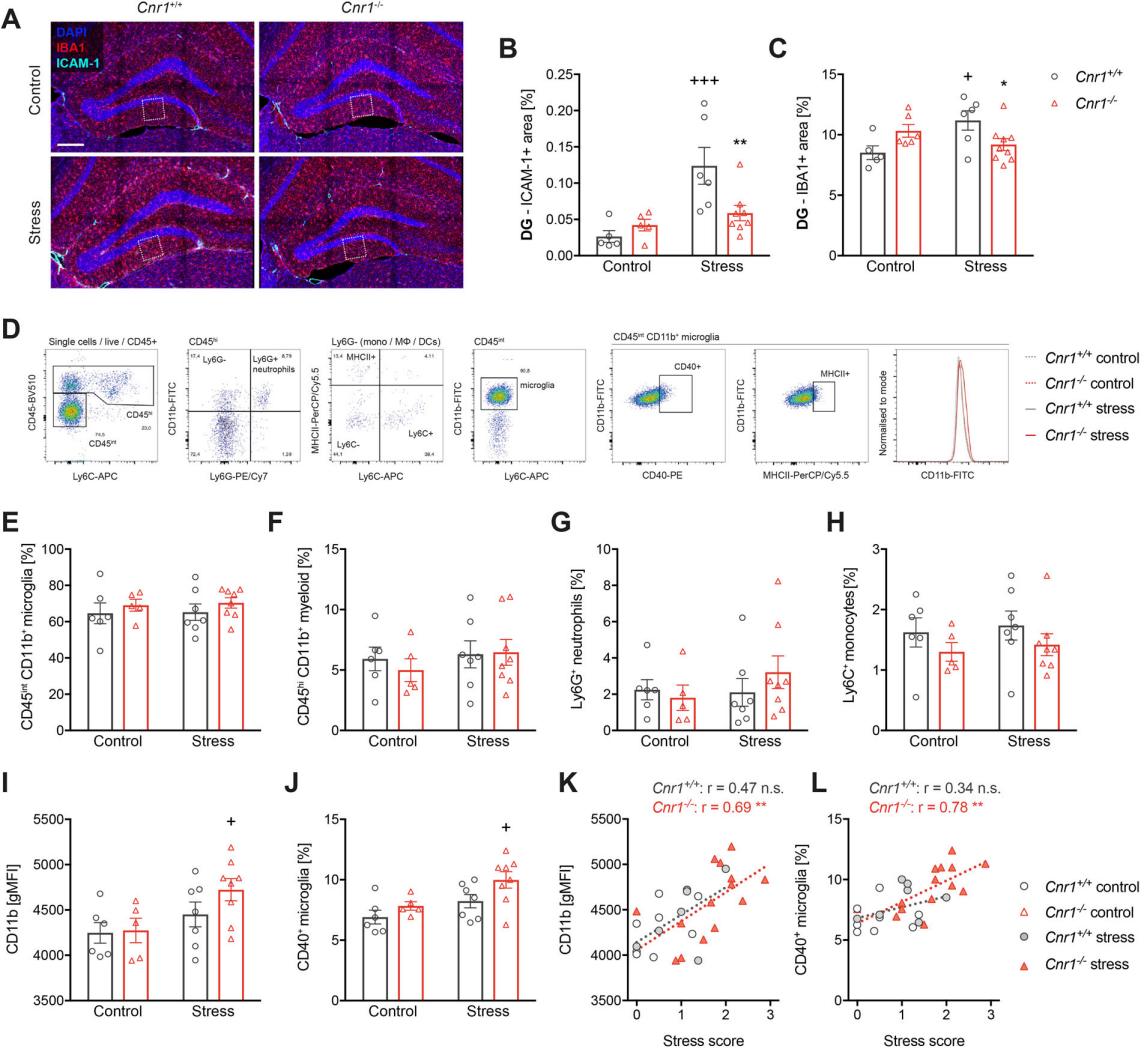
Mild CSDS does not cause infiltration of myeloid cells to the brain, but alters microglial activation in *Cnr1*^−/−^ mice. **A** Immunofluorescent staining of microglia (IBA1, red) and ICAM-1 (cyan) after mild CSDS. Single channels can be seen in Supplementary Fig. 4. Maximum projections of 9 µm z-stacks acquired with 20x magnification are shown (scale bar = 100 µm). **B** Quantification of ICAM-1 + area in the DG (stress: *F*_(1,20)_ = 13.17, *p* = 0.0017; stress x genotype interaction: *F*_(1,20)_ = 6.62, *p* = 0.018) and **C** IBA1 + area in the DG (stress x genotype interaction: *F*_(1,22)_ = 9.23, *p* = 0.006). **D** Representative gating strategy for flow cytometry of brain myeloid cells (pre-gated for single CD45^+^ cells). **E**–**H** Relative frequencies of brain myeloid cells after mild CSDS: **E** microglia (CD45^int^ CD11b^+^ Ly6G^-^) and **F** infiltrated myeloid cells (CD45^hi^ CD11b^+^), which were further separated into **G** neutrophils (CD11b^+^ Ly6G^+^) and **H** Ly6C^hi^ monocytes (CD11b^+^ Ly6G^-^ Ly6C^hi^) (no significant main effects for any). **I** Geometric mean fluorescence intensity (gMFI) of CD11b on microglia (stress: *F*_(1, 22)_ = 6.24, *p* = 0.021)**. J** Percentage of CD40^+^ microglia (stress: *F*_(1, 22)_ = 8.21, *p* = 0.009; genotype: *F*_(1, 22)_ = 4.89, *p* = 0.038). Data were analysed by two-way ANOVA followed by Bonferroni post-hoc test for multiple comparison. Post-hoc significances shown in graph: *p < 0.05 vs*. Cnr1*^+/+^ of the same condition; +*p* < 0.05 vs. control of the same genotype. **K**, **L** Spearman correlation of microglial activation markers with behavioural stress scores: **K** CD11b gMFI (*Cnr1*^+/+^: *r* = 0.47, *p* = 0.082; *Cnr1*^−/−^: *r* = 0.69, *p* = 0.008; both genotypes: *r* = 0.63, *p* = 0.0002). **L** CD40^+^ microglia (*Cnr1*^+/+^: *r* = 0.34, *p* = 0.216; *Cnr1*^−/−^: *r* = 0.78, *p* = 0.001; both genotypes: *r* = 0.65, *p* = 0.0001).

In rodents, social defeat and consequent anxiety-like behaviour is associated with the recruitment of myeloid cells to the brain, facilitated by increased expression of intracellular adhesion molecule 1 (ICAM-1) on neurovascular endothelial cells^9,49^. After mild CSDS, we observed increased neurovascular ICAM-1 expression in the brain of *Cnr1*^*+/+*^, but not *Cnr1*^−/−^ mice (Fig. 4A, B and Supplementary Fig. 4). Yet, using flow cytometry, we could not detect increased infiltration of peripheral myeloid cells (Fig. 4D–H). However, surface expression of activation markers CD11b and CD40 on microglia was altered after stress (Fig. 4I, J). In *Cnr1*^−/−^, but not *Cnr1*^+/+^ mice, stress significantly increased surface expression of CD11b on microglia (Fig. 4I) and the frequency of CD40 + microglia (Fig. 4J). These parameters were further positively correlated with the behavioural stress score in *Cnr1*^−/−^ mice or when analysing all samples independent of the genotype (Fig. 4K, L). A similar pattern was observed for MHCII + microglia as well (data not shown).

### Mild CSDS alters microglia morphology in a CB1-dependent manner

To further investigate how CB1-deficiency alters microglial function after mild CSDS, we analysed IBA1 immunoreactivity in stress-responsive brain regions. In the dentate gyrus (DG), IBA1 + area was increased by stress in *Cnr1*^+/+^, but not in *Cnr1*^−/−^ mice (Fig. 4C). Similar, but less pronounced effects were seen in the CA1 region, amygdala and mPFC (Supplementary Fig. 4). To confirm that the observed effects were based on microglia and not infiltrated monocytes/macrophages (which are also IBA1 + ), sections were co-stained for IBA1 and TMEM119, a microglia-specific marker that is not expressed by perivascular, meningeal or peripheral macrophages^50^. IBA1 + and TMEM119 + area in the DG were highly correlated with each other and the stress-induced changes in TMEM119 + area were similar to those observed for IBA1 (Supplementary Fig. 5), supporting the finding that mild CSDS did not induce infiltration of peripheral monocytes, but affected brain-resident microglia.

**Fig. 5:**
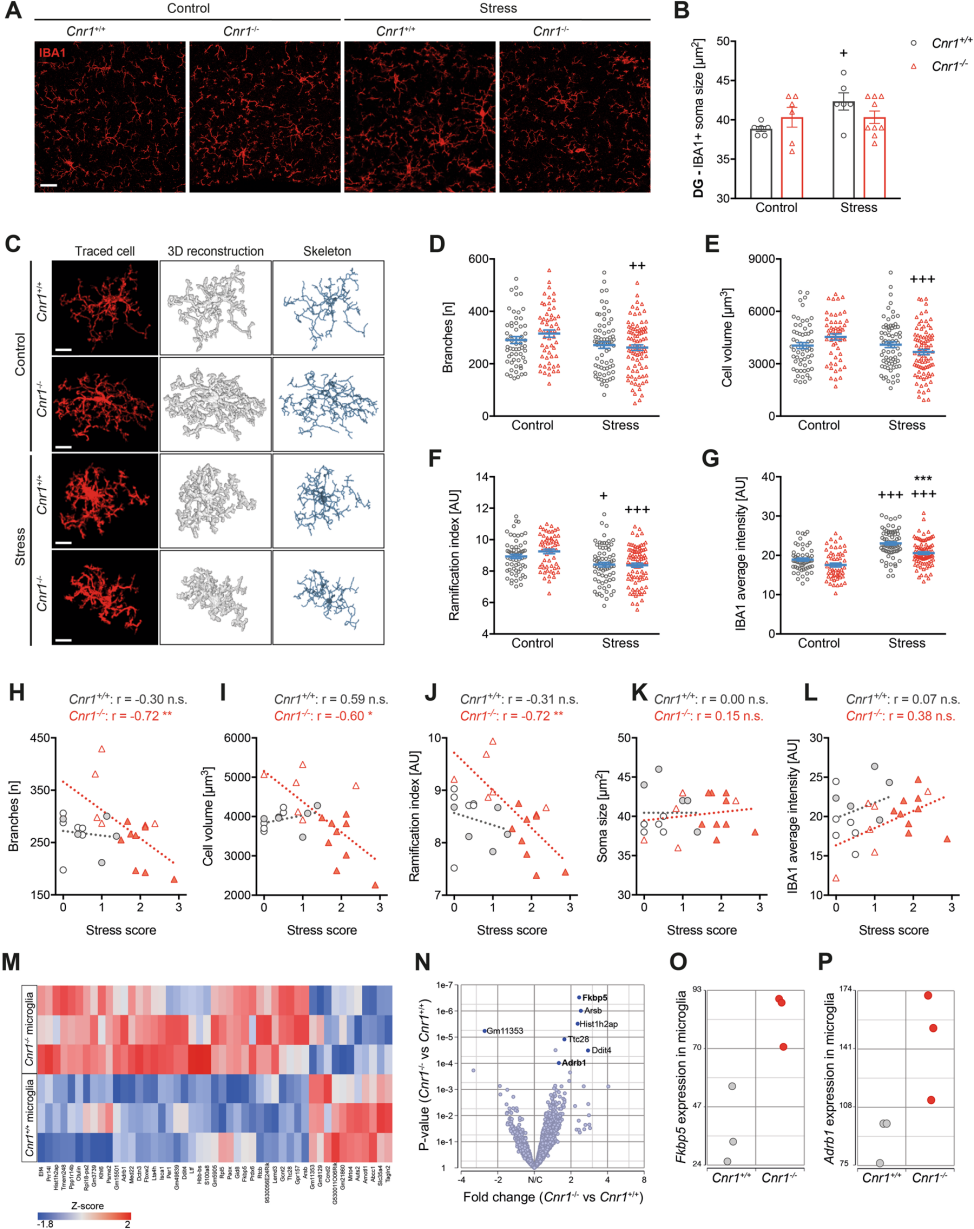
Microglia of *Cnr1*^−/−^ mice show a de-ramified morphology after mild CSDS and altered expression of stress-related genes. **A** Immunofluorescent staining of microglia (IBA1, red) in the molecular layer of the DG (region indicated by white boxes in Fig. 4A). Maximum projections of 5 µm z-stacks acquired with 63x magnification are shown (scale bar = 20 µm). Note that images are derived from IBA1/ICAM-1-co-stainings. Merged channels can be seen in Supplementary Fig. 4. **B** Microglia soma size, quantified manually (stress: *F*_(1,23)_ = 3.51, *p* = 0.074; stress x genotype interaction: *F*_(1,23)_ = 3.51, *p* = 0.074). **C** Microglia morphology was analysed from 40–50 µm z-stacks, using an ImageJ-based analysis tool. Representative images of individually traced microglia cells, 3D reconstructions and skeletons of the reconstructed cells are shown (scale bar = 10 µm). **D**–**G** Quantification of morphology of individual microglial cells from 60–87 cells per group: **D** Number of branches per cell, determined from skeletons (stress: *F*_(1,269)_ = 8.465, *p* = 0.0039); **E** cell volume determined from 3D reconstructed cells (stress: *F*_(1,265)_ = 5.712, *p* = 0.0175; stress x genotype interaction: *F*_(1,265)_ = 7.282, *p* = 0.0074); **F** ramification index, a unit-free parameter describing the complexity of the cellular shape, determined from 3D reconstructed cells (stress: *F*_(1,269)_ = 24.0, *p* < 0.0001); **G** average intensity (mean grey) of IBA1 immunoreactivity on traced cells (stress: *F*_(1,269)_ = 76.4, *p* < 0.0001; genotype: *F*_(1,269)_ = 22.24, *p* < 0.0001). Data were analysed by two-way ANOVA followed by Bonferroni post-hoc test for multiple comparison. Post-hoc significances shown in graph: *p < 0.05 vs*. Cnr1*^+/+^ of the same condition; +*p* < 0.05 vs. control of the same genotype. **H**–**L** Spearman correlation was performed for mean morphological values of each animal and the respective behavioural stress score: **H** number of branches per cell (*Cnr1*^+/+^: *r* = −0.29, *p* = 0.38; *Cnr1*^−/−^: *r* = −0.72, *p* = 0.003; both genotypes: *r* = −0.40, *p* = 0.044); **I** cell volume (*Cnr1*^+/+^: *r* = 0.59, *p* = 0.061; *Cnr1*^−/−^: *r* = −0.60, *p* = 0.019; both genotypes: *r* = −0.28, *p* = 0.168); **J** ramification index (*Cnr1*^+/+^: *r* = −0.31, *p* = 0.357; *Cnr1*^−/−^: *r* = −0.72, *p* = 0.004; both genotypes: *r* = −0.48, *p* = 0.012); **K** microglia soma size (*Cnr1*^+/+^: *r* = −0.02, *p* = 0.959; *Cnr1*^−/−^: *r* = 0.09, *p* = 0.739; both genotypes: *r* = 0.09, *p* = 0.667); and **L** IBA1 mean intensity (*Cnr1*^+/+^: *r* = 0.07, *p* = 0.839; *Cnr1*^−/−^: *r* = 0.38, *p* = 0.166; both genotypes: *r* = 0.18, *p* = 0.371). **M**–**P** Transcriptome analysis was performed using 3′ mRNA sequencing of microglia (enriched CD11b + cells) isolated from stress-naïve *Cnr1*^*+/+*^ and *Cnr1*^−/−^ mice. Differentially expressed (DE) genes between *Cnr1*^−/−^ vs. *Cnr1*^*+/+*^ microglia were determined using one-factorial ANOVA and FDR step-up for multiple testing correction. **M** Heatmap visualisation of the top DE genes with *p* < 0.01 and a fold change (FC) of >1.5; **N** Volcano plot of DE genes, with significantly regulated genes (FDR adjusted *p* < 0.1, FC > 1.5) highlighted in dark blue; **O**, **P** Example plots of normalised counts for significant DE genes in *Cnr1*^−/−^ vs. *Cnr1*^*+/+*^ microglia **O** *Fkbp5* and **P** *Adrb1*.

The number of IBA1 + cells in the DG was not different between the groups (Supplementary Fig. 4), suggesting that the difference in IBA1 + area was due to altered morphology or IBA1 expression. Therefore, microglia in the molecular layer of the DG were analysed in more detail, revealing different stress-induced morphological rearrangements in *Cnr1*^−/−^ compared to *Cnr1*^+/+^ mice (Fig. 5). In *Cnr1*^+/+^ mice, stress caused a significant increase in cell soma size, which was not observed in *Cnr1*^−/−^ mice (Fig. 5B). In contrast, 3D reconstruction of individual cells showed that stress specifically altered microglial arborisation in *Cnr1*^−/−^ mice (Fig. 5C–F). Compared to control conditions, the number of branches (Fig. 5D) and cell volume (Fig. 5E) were reduced after stress in *Cnr1*^−/−^ microglia. Control *Cnr1*^−/−^ microglia were slightly larger and more ramified than control *Cnr1*^+/+^ microglia, however these differences did not reach statistical significance in post-hoc comparisons. Similar effects were observed for other morphological parameters and to a lesser extend also for microglia within the CA1 region (Supplementary Fig. 6). The ramification index, a measure for the complexity of the cellular shape, was significantly reduced by stress in both genotypes (Fig. 5F). This was likely caused by reduced branching in *Cnr1*^−/−^ mice and by enlarged cell soma in *Cnr1*^+/+^ mice, respectively. Furthermore, IBA1 intensity on individual microglia was significantly increased by stress in both genotypes and higher on microglia of stressed *Cnr1*^+/+^ compared to stressed *Cnr1*^−/−^ mice (Fig. 5G). Correlation of morphological parameters with the behavioural stress score showed that microglial changes were associated with the behaviour in *Cnr1*^−/−^, but not in *Cnr1*^*+/+*^ mice (Fig. 5H–J). Thus, *Cnr1*^−/−^ mice showing strong behavioural deficits had the lowest microglial complexity (e.g. number of branches, cell volume, ramification index). In contrast, parameters that were altered by stress in *Cnr1*^+/+^ mice were not correlated with the behavioural phenotype, namely IBA1 intensity and soma size (Fig. 5K, L).

### Microglia of Cnr1^−/−^ mice show altered expression of stress-related genes

To explore the molecular mechanisms linking CB1 signalling to microglial responses to stress, we isolated microglia (enriched CD11b^+^ cells) from naïve *Cnr1*^+/+^ and *Cnr1*^−/−^ mice and performed gene expression analysis using 3′ mRNA sequencing. On a global level, *Cnr1*^−/−^ microglial transcriptomes did not fundamentally differ from those of *Cnr1*^+/+^ mice, indicated by no clear clustering by genotype in either PCA, tSNE or hierarchical clustering (Supplementary Fig. 7). However, differential expression analysis revealed that several genes were altered in *Cnr1*^−/−^microglia, with 414 genes reaching nominal significance (*p* < 0.05) (Fig. 5M and Supplementary Table 2), of which seven genes remained significant after correcting for multiple testing (FDR adjusted *p* < 0.1) and having a fold change of at least 1.5 (Fig. 5N). Interestingly, among these were two genes involved in stress signalling and depressive disorders, namely *Fkbp5* (encoding FK506 binding protein 5 (FKBP5), a regulator of glucocorticoid receptor (GR) activity) and *Adrb1* (encoding the beta-1 adrenergic receptor (β_1_-AR)). Both were upregulated in microglia of *Cnr1*^−/−^ mice (Fig. 5O–P). Among nominal significantly upregulated genes were other GR-responsive genes, such as *Nfkbia*, encoding the NFκB inhibitor IκBα, and *Tsc22d3*, encoding the glucocorticoid-induced leucine zipper (GILZ) (Supplementary Table 2).

## Discussion

CB1 signalling has been associated with altered stress responsiveness and the aetiology of major depressive disorders. Our findings confirm that CB1 signalling dampens the severity of the behavioural responses upon exposure to mild chronic stress and modulates the HPA axis activity. In addition, our findings suggest that lack of CB1 signalling during chronic stress causes insufficient GC signalling. Furthermore, CB1 signalling modulates the stress responsiveness of brain-resident microglia, indicated by altered expression of stress-related genes at baseline and increased expression of activation markers as well as changes in morphology after stress in *Cnr1*^−/−^ mice. Interestingly, microglial parameters correlate with the severity of the behavioural phenotype, thus implicating endocannabinoid-mediated modulation of microglia in the development of stress-related pathologies.

In a standard model of CSDS^44^, we observed a very severe phenotype of *Cnr1*^−/−^ mice, showing mortality rates of ~50%—compared to 10% in *Cnr1*^+/+^ mice. Mice lacking DAGLα, the main 2-AG synthesising enzyme, also showed a mortality rate of 30%. It is well known that the *Cnr1*^−/−^ line has an increased spontaneous mortality rate, although the underlying pathophysiology remains unclear^43^. The observations in this study now suggest that the increased spontaneous mortality is linked to the increased stress responsiveness of the mouse line. Since the death of *Cnr1*^−/−^ mice during standard CSDS was reminiscent of sudden cardiac death, we analysed their heart activity during CSDS. *Cnr1*^−/−^ mice did not show signs of myocardial infarction or ventricular fibrillation (the most common cause of sudden cardiac death), but were bradycardic and developed total AV-blocks before death. This could possibly be related to cardiovascular effects of CB1 signalling via the SNS^51,52^ and/or vagal over-activation in response to excessive SNS activity, which has been associated with the occurrence of AV block^53^. CB1 signalling thus seems especially indispensable in situations of severe stress and lack of it may impose a serious risk, possibly related to cardiovascular complications.

Social defeat stress in rodents produces behavioural changes that are relevant to symptoms of human psychiatric disorders, such as depression or posttraumatic-stress disorder (PTSD). In the standard CSDS paradigm applied here, all mice that survived the procedure showed clear social avoidance, which is typically used to assess stress-susceptibility^44^. We and others have however observed that social avoidance is not correlated with other stress-related behaviours, including anxiety and anhedonia^54,55^. In later experiments, we therefore decided to measure stress-susceptibility by calculating a behavioural stress score that incorporates performances from all tests. In the mild version of the CSDS protocol, significant behavioural changes were only observed in *Cnr1*^−/−^ mice, in individual tests as well as the overall stress score. This demonstrates that a disruption of CB1 signalling has adverse consequences even under mild stress conditions that do not affect wild type mice.

It is commonly assumed that sustained elevation of GC levels contributes to chronic stress-related pathologies. However, stress-related neuropsychiatric disorders are often associated with insufficient GC signalling^56^. For example, patients suffering from PTSD or chronically stressed subjects often show low CORT responses and overall blunted HPA axis activity^57–59^. Another common observation in chronically stressed individuals is a flattening of diurnal CORT patterns, with lower secretion at wakening and during the active phase, but higher levels during the inactive phase^57,60–62^. The effects of chronic stress on diurnal CORT are possibly mediated by altered endocannabinoid/CB1 signalling. A progressive increase of amygdalar 2-AG levels during chronic stress is thought to suppress CORT production in anticipation of the re-encountered stressor, while a decrease in AEA likely induces basal hypersecretion of CORT during the light phase^63^. These adaptive changes cannot take place in the absence of CB1 receptors. In line with this, plasma CORT levels at the beginning of the dark phase, i.e. the time of expected stress exposure, were higher in stressed *Cnr1*^−/−^ mice compared to stressed *Cnr1*^+/+^ mice. In contrast, cumulative daily CORT levels were increased by stress only in *Cnr1*^+/+^, likely caused by the above mentioned AEA-mediated hypersecretion of CORT during the light phase. Thus, lack of CB1 signalling during chronic stress causes dysregulation of HPA axis activity, overall leading to reduced GC synthesis.

GCs produced during repeated stress stimulate the egress of monocytes and neutrophils from the bone marrow into the circulation^9^. Furthermore, stress-induced adrenergic signalling stimulates the proliferation of HSCs and subsequent inflammatory monocyte and neutrophil production^6^. Both mechanisms involve the chemokine CXCL12, which inhibits HSC proliferation and migration and retains cells within the bone marrow^64,65^. Mild CSDS slightly increased TH staining intensity (an indirect measure of NE synthesis) and reduced *Cxcl12* gene expression in the bone marrow. However, we generally did not see increased monocyte frequencies in the bone marrow or circulation after mild CSDS. Only in the spleen, a significant stress effect with respect to Ly6C^hi^ monocytes was observed. Albeit not significant, stressed *Cnr1*^−/−^ mice had slightly higher Ly6C^hi^ monocyte frequencies in the bone marrow than *Cnr1*^+/+^ mice but lower numbers in the blood. This could indicate that lower GC levels in stressed *Cnr1*^−/−^ mice prevented the release of monocytes into the circulation. Additionally, *Cnr1*^−/−^ mice showed reduced expression of CCR2 on monocytes, which is required for their egress from the bone marrow^66^. With the exception of neutrophils, which were increased by mild CSDS in the bone marrow of *Cnr1*^−/−^ mice, the mild CSDS protocol overall produced only moderate changes in peripheral myeloid cell populations.

In other studies, peripheral monocytes were shown to be recruited to the brain neurovascular space and possibly enter into the brain parenchyma^9,13,49^, which could be prevented by pharmacological treatment with a CB1/CB2 agonist and thereby reverse social defeat-induced anxiety^42^. The recruitment of peripheral cells is mediated by increased ICAM-1 expression by brain endothelial cells. In *Cnr1*^+/+^ mice, the mild CSDS stress protocol indeed caused an increase in neurovascular ICAM-1 in stress-related brain regions. Nonetheless, we did not detect increased numbers of monocytes or other myeloid cells in the brain. Since we also did not observe significantly higher monocyte frequencies in peripheral tissues in these mice, it is likely that the mild version of CSDS was not sufficient to induce pronounced myelopoiesis and consequently no monocyte trafficking to the brain. The induction of reactive brain endothelium and peripheral myelopoiesis therefore seem to be mediated by independent pathways. Indeed, stress-induced myelopoiesis in the bone marrow depends on adrenergic signalling^6,9,67^, while expression of ICAM-1 is dependent on CORT released during chronic stress^9^. Interestingly, neurovascular ICAM-1 expression was only induced in *Cnr1*^+/+^, but not in *Cnr1*^−/−^ mice, further supporting the finding that lack of CB1 signalling results in reduced GC signalling during chronic stress.

In contrast to the relatively weak effects on peripheral myeloid cell populations, mild CSDS did significantly affect brain-resident microglia and these changes were correlated to the degree of behavioural stress-susceptibility. Several studies have previously demonstrated a causal relationship between microglia and stress-related pathologies. As such, inhibition of microglial activation by minocycline treatment was shown to prevent chronic stress-induced neuroinflammation and the development of anxiety- and depressive-like behaviour^68–72^. Similarly, depletion of microglia during repeated social defeat protected animals from stress-induced behavioural deficits^73,74^. Here, we show that surface expression of activation markers on microglia was significantly upregulated after mild CSDS only in *Cnr1*^−/−^ mice. Interestingly, expression of these markers was positively correlated with behavioural stress scores and was accordingly highest in stressed *Cnr1*^−/−^ mice. Additionally, stress caused an increase in microglial staining (IBA1 + area) in the hippocampus, which has been reported in several other studies^12,14,75^. Again, this effect was only seen in *Cnr1*^+/+^, but not in *Cnr1*^−/−^ mice. These findings resemble those seen in mice depleted for CORT during social defeat^9^. An increase in IBA1 + area is often considered a sign of microglial activation, although it can be the result of different factors, such as increased numbers of microglia, altered morphology, or increased IBA1 expression. Detailed analysis of hippocampal microglia after mild CSDS revealed that the different regulation of IBA1 + area between *Cnr1*^+/+^ and *Cnr1*^−/−^ mice in response to stress was in fact caused by separate mechanisms. In *Cnr1*^+/+^ mice, the increase in IBA1 + area was a result of increased IBA1 expression and slightly enlarged cell bodies. In contrast, the absence of a stress-induced increase of IBA1 + area in *Cnr1*^−/−^ mice was related to morphological rearrangements of microglia to a smaller, de-ramified state. Interestingly, only the morphological changes in *Cnr1*^−/−^ microglia, which were mostly related to the branching of cells, were correlated to the behavioural stress score.

Microglial morphology changes are typically observed under neuroinflammatory conditions. However, several previous studies suggest that stress-induced changes in microglial ramification can occur independent of clear inflammatory changes^76,77^. In line with that, we did not observe induction of hippocampal gene expression of pro-inflammatory cytokines IL-6, IL-1β or TNFα after mild CSDS in either genotype (data not shown). Alternatively, stress-induced morphological rearrangements might be mediated through endocrine signals or changes in neuronal activity. Interestingly, among the few genes that were differentially expressed in microglia isolated from naïve *Cnr1*^+/+^ and *Cnr1*^−/−^ mice were two candidates involved in GC and adrenergic signalling, respectively. The top hit was *Fkbp5*, a co-chaperone of Hsp90 negatively regulating GR sensitivity and nuclear translocation, which has been implicated as a genetic risk factor in depressive disorders^78^. The expression of *Fkbp5* is induced by GCs and regulated through epigenetic mechanisms^79^. Increased expression of *Fkbp5* in microglia of *Cnr1*^−/−^ mice thus suggest enhanced baseline GC signalling, which is supported by a slight increase in other GR-responsive genes *Nfkbia* and *Tsc22d3*. On the other hand, high FKBP5 levels are thought to result in GC resistance and impaired negative feedback control, possibly making *Cnr1*^−/−^ microglia less sensitive to the effects of GCs, including their effect on microglial ramification. Analogous to our observations, increasing GC levels in young mice caused hyper-ramification of microglia in the hippocampus, while reducing microglial sensitivity to GCs in old mice further aggravated age‐related microglial de-ramification^80^. An interaction between GC and adrenergic signalling in microglial stress responses was indicated by experiments demonstrating that *Fkbp5* expression was reduced in microglia of socially defeated mice, which could be rescued by β-AR antagonist pre-treatment^75^. Generally, neuronal NE is thought to suppress microglial activation (e.g. pro-inflammatory cytokine expression and microglial ramification and surveillance) through binding to microglial β-ARs^81^. However, microglial activation induced by acute restraint stress or repeated social defeat could be prevented by blockade of β_1_/β_2_-ARs^75,82^. Here we show an increased expression of the gene encoding β_1_-AR in *Cnr1*^−/−^ microglia, which could enhance their responsiveness to NE released during stress. Together, these findings suggest that the lack of CB1 receptors alters stress hormone signalling in microglia, making them vulnerable even under mildly stressful conditions. Especially the connection between CB1 and *Fkbp5* in microglia warrants further analysis in future studies. Since microglia themselves hardly express CB1 receptors, its effect on stress signalling in microglia is likely mediated indirectly through neuronal CB1 signalling. In a study using a slightly different CSDS protocol, it was shown that CB1 receptors on different neuronal populations regulate different aspects of the behavioural consequences of stress^83^. While it was beyond the scope of the current study, it will be important to elucidate which cell type or subtype of CB1-expressing cells is responsible for mediating the effects of stress on microglial function and how they influence specific aspects of stress-related pathologies.

In summary, this study provides clear evidence that CB1 signalling protects the body from the physical and emotional harms of social stress. Even under mildly stressful conditions, which do not result in the development of maladaptive behaviours in wild type mice, CB1-deficiency greatly increases the proportion of mice that show a stress-related behavioural phenotype. The fact that stressed *Cnr1*^−/−^ mice showed a clear behavioural phenotype without strong changes in peripheral myeloid cells and no recruitment of monocytes to the brain argues against a crucial involvement of those cells. In contrast, different parameters of microglial function were changed after mild CSDS and correlated with the behavioural susceptibility—especially in *Cnr1*^−/−^ mice. Finally, we show that CB1-deficiency alters the expression of stress-related genes in microglia, providing an explanation for the differential response of *Cnr1*^−/−^ microglia to stress and further supporting the important role of microglia in stress-related pathologies.

## Supplementary information

Supplementary Material

Supplementary Figure 1

Supplementary Figure 2

Supplementary Figure 3

Supplementary Figure 4

Supplementary Figure 5

Supplementary Figure 6

Supplementary Figure 7

Supplementary Table 1

Supplementary Table 2
