# Supplementary material for: Cannabinoid receptor 1 signalling modulates stress susceptibility and microglial responses to chronic social defeat stress
Source: Transl Psychiatry. 2021 Mar 15;11:164. doi: 10.1038/s41398-021-01283-0 (PMC7961142; doi:10.1038/s41398-021-01283-0)
Supplement: Supplementary file 1 — Supplementary Material [file 41398_2021_1283_MOESM1_ESM.docx]

**Supplementary methods**

***Mice***

In this study, B6.cgCnr1 tm1Zim (constitutive CB1 knockout) and B6.cg*Dagla* tm1.2Zim (constitutive DAGLa knockout) on a C57BL/6J background were used. C57BL/6J mice were originally obtained from a commercial breeder (Charles River) and bred at the animal facility of the University of Bonn. B6.cgCnr1 tm1Zim mice were generated by disrupting the *Cnr1* coding region with a neomycin cassette, using homologous recombination in embryonic stem cells ^1^. B6.cgDagla tm1.2Zim were generated by inserting two loxP sites flanking exon 1 of the *Dagla* gene, allowing Cre-mediated excision of the exon. Constitutive *Dagla*^-/-^ mice were obtained by breeding homozygous *Dagla* floxed (*Dagla*^fl/fl^) mice with Tg(Pgk1-cre)1Lni mice ^2^. Mutant mice were backcrossed to C57BL/6J mice for more than ten generations. For this study, B6.cgCnr1 tm1Zim mice were bred mainly using heterozygous breeding pairs (*Cnr1*^+/-^ x *Cnr1*^+/-^). Homozygous wild type (*Cnr1*^+/+^) and homozygous CB1 knockout (*Cnr1*^-/-^) littermates were used for experiments. B6.cgDagla tm1.2Zim mice were bred in homozygous *Dagla*^-/-^ and *Dagla*^fl/fl^ breeding pairs. Mice were genotyped by PCR using the following primers:
CB1 WT fwd: 5’-TGTGTCTCCTGCTGGAACCAACGG-3’, CB1 KO fwd: 5’‑TCTCTCGTGGGATCATTGTTTTTCTCTTGA‑3’; CB1 rev: 5’‑CTCCTGGCACCTCTTTCT-CAGTCA‑3’; DAGLa KO fwd: 5’‑TAGCTTAGCCCCCATGTGA‑3’; DAGLa rev: 5’‑CCCAGT-AGCCACAGACAT‑3’; DAGLa WT: 5’‑GAGATGGGTTCCACCTCCTT‑3’. All experiments followed the guidelines of the German Animal Protection Law and were approved by the Local Committee for Animal Health, LANUV NRW (84-02.04.2015.A192, 84-02.04.2013.A488).

***Chronic social defeat stress cohorts***

Calculation of sample sizes for behavioural analysis after CSDS was performed based on previous experience within the group using a priori analysis in the G*Power 3.1 software (ANOVA with fixed effects, special, main effects and interactions; f = 0.45, α = 0.05, β = 0.8, df = 1, number of groups = 4). Standard CSDS experiments presented here were performed in two separate cohorts of *Cnr1* mice (cohort one: *Cnr1*^+/+^ control n = 6, stress n = 8; *Cnr1*^-/-^ control n = 4, stress n = 6; cohort two: *Cnr1*^+/+^ control n = 4, stress n = 14; *Cnr1*^-/-^ control n = 4, stress n = 9). In cohort two, *Cnr1*^-/-^ mice were excluded on day four of the CSDS protocol, due to high mortality (6 out of 9). Experiments with *Dagla* mice were performed in one cohort (n = 10 per group). Analysis of heart activity during standard CSDS was analysed in a separate cohort of *Cnr1*^-/-^ mice (n = 7). Mild CSDS experiments were performed successively in three cohorts, using the same setup and CD1 aggressor mice (cohort one: *Cnr1*^+/+^ control n = 6, stress n = 6; *Cnr1*^-/-^ control n = 6, stress n = 9; cohort two: *Cnr1*^+/+^ control n = 2, stress n = 3; *Cnr1*^-/-^ control n = 3, stress n = 3; cohort three: *Cnr1*^+/+^ control n = 5, stress n = 5; *Cnr1*^-/-^ control n = 4, stress n = 4). Behavioural and endocrine data was pooled from all cohorts. Cohort one was used for immunohistochemical analysis of microglia and cohort one and two for flow cytometry. Cohort three was used for bone marrow immunohistochemistry and qPCR. Two control cages, i.e. two *Cnr1*^-/-^ and two *Cnr1*^+/+^ mice, had to be excluded from later analyses, due to highly deviant baseline behaviours. Animals were randomly assigned to the groups while trying to balance the distribution of littermates between control and stress. Control animals housed in the divided cages were always from different stock cages and whenever possible housed in pairs of one *Cnr1*^+/+^ and one *Cnr1*^-/-^ animal.

***Behavioural analysis after CSDS***

Behavioural analysis was performed during the active phase of the animals in a sound-isolated room and performed using automated video tracking software. The investigator was not blinded to the group or genotype. The order of animals during the tests was chosen randomly.

***Open field test***

Anxiety behaviour and locomotion were analysed using the open field test**.** Mice were tested in an open field box (44 cm x 44 cm) with 150 lux illumination. Movement of mice was recorded for 10 minutes using EthoVision XT software (Noldus Information Technology Inc.).

***Social avoidance test***

Social avoidance behaviour was analysed on the last day of CSDS, approximately 2 hours after the last defeat. The test was divided into two trials of 2.5 minutes each. During the first trial, test mice were placed into an open field box containing an empty metal grid cage (no target). For the second trial, the cage was replaced by a cage containing a novel CD1 mouse (target). Movement was recorded using EthoVision XT. Interaction was measured as time spent in close proximity to the cage (nose-point). Boxes were disinfected after every test to remove residual olfactory cues from CD1 mice.

***Sucrose preference test***

To analyse hedonic behaviour, mice had free access to two bottles for 24 hours, one containing water and the other containing 1% sucrose solution. During the test, positions of the bottles were changed at least once to avoid side-bias. Consumption was measured by weighing the bottles and preference was calculated as percent sucrose consumption of total fluid consumption.

***Nestlet test***

Nest building provides shelter from predators, helps conserving body temperature and in laboratory mice is considered a home-cage activity related to social behaviour ^3,4^. In the nestlet shredding test, mice were provided with a pre-weighed nestlet made of compressed cotton. After 1 h, the remaining intact nestlet was removed and weighed to calculate the amount of material used.

***Telemetric measurement of heart activity***

To analyse cardiac activity of mice during CSDS, long-term electrocardiograms (ECG) were recorded in *Cnr1^-/-^* mice (n = 7). Two weeks before starting the CSDS paradigm, mice were implanted with telemetric ECG-transmitters specifically made for small rodents (Data Science International, St. Paul, USA). For implantation, mice were anaesthetised by i.p. injection of xylazin hydrochloride (16 mg/kg) and ketamin hydrochloride (100 mg/kg) and received s.c. injections of the analgesic carprofen (5 mg/kg) once during and for three consecutive days after the surgery. Anaesthetised mice were fixed on a warming plate (37°C) and eyes were protected from drying with eye ointment (Bepanthen®). After shaving the fur, a small incision of the skin was prepared between the scapulae and the transmitter implanted subcutaneously. Additionally, two electrode wires were implanted subcutaneously at the left-lateral and anterior thoracic wall. The wound was closed with prolene threads. After the surgery, mice behave normally with respect to circadian rhythm, feeding, and grooming. The wound is usually healed after approximately one week, but since mice were exposed to physical contact with CD1 aggressor mice during CSDS, a recovery period of two weeks was applied in this study. Mice were subjected to the standard CSDS paradigm and heart activity was recorded during the 10-day period (no recording during the 10 min daily stress exposure). Measurements were performed by telemetric recording plates that were placed below the cages of the mice and thus did not require any further manipulation on the animal.

***Tissue collection and isolation of cells***

One day after the last defeat, mice were sacrificed and perfused with PBS. Blood was collected in EDTA-tubes by cardiac puncture. For RNA isolation, organs were snap-frozen and stored at -80°C. For immunohistochemistry, organs were post-fixed in 4% formaldehyde at 4°C, followed by cryoprotection in 20% sucrose. Fixed organs were snap-frozen and stored at -80°C. For flow cytometry, organs were transferred into PBS or corresponding medium and kept on ice. Bone marrow cells were flushed out from tibia with PBS. Single cell suspensions were prepared and filtered through a 70 µm cell strainer. Cells were centrifuged (300 x g, 7 min, 4°C) and resuspended in FACS buffer (PBS + 2 % FCS). Spleen tissue was digested with DNase I / Collagenase type VIII (Sigma-Aldrich) at 37°C for 45 minutes. Homogenates were filtered through a 70 µm cell strainer, washed with FACS buffer, centrifuged (300 x g, 7 minutes, 4°C) and washed. For isolation of brain mononuclear cells, one hemisphere was collected in DMEM, high glucose (Gibco) + 10% FCS. Tissue was homogenised and digested with 20 µg/ml DNase I + 20 µg/ml Collagenase/Dispase (Roche) at 37°C for 45 minutes. Single cell suspensions were prepared and filtered through a 70 µm cell strainer, washed and centrifuged (300 x g, 7 minutes, 4°C). Separation of leukocytes was performed using an isotonic 40/80% Percoll gradient centrifugation. Cells resuspended in 40% Percoll were sublayered with 80% Percoll and centrifuged at 1000 x g, 25 minutes at RT. After centrifugation, leukocytes were collected at the 40/80% interface, washed and filtered through gauze.

***Cell culture of splenocytes***

Isolated splenocytes were resuspended in splenocyte culture medium (RPMI 1640, supplemented with 10% FCS, 1% penicillin/streptomycin, 1% L-glutamine) and seeded in 12-well plates at a density of 1 x 10^6^ cells/ml. Cells were either untreated or stimulated with 1 µg/ml LPS from *E. coli O127:B8* overnight (one well per animal). After overnight culture, cells and medium were collected and centrifuged (300 x g, 5 min, 4°C). Supernatants were transferred to a fresh tube, snap-frozen in liquid nitrogen and stored at - 20°C. Cells were resuspended in FACS buffer and stained for flow cytometric analysis.

***Brain IBA1 / ICAM-1 immunohistochemistry, image acquisition and analysis***

For microglia analysis, free-floating 60 µm coronal sections were prepared from frozen hemispheres. Sections were blocked in 10% normal donkey serum (NDS) in PBS + Triton-X 0.5% for 4 hours, followed by overnight incubation at 4°C with primary antibodies against IBA1, ICAM-1, and CD45 diluted in 5% NDS in PBS-T 0.1% (see Supplementary Table 1 for details). After washing, sections were stained with secondary antibodies for 4 hours. Nuclei were stained with DAPI and washed sections mounted in Fluoromount-G® (SouthernBiotech). Sections were imaged using a Leica TCS SP8 confocal microscope. For overview pictures, images were acquired with a 20x objective (NA = 0.75), pinhole = 1 AU, 0.28 µm/pixel. Two to three sections were imaged per animal for each region. Images were analysed using Fiji (ImageJ 2.0.0). For measurement of IBA1- or ICAM-1-positive area, images were thresholded to exclude background signal. For 3D morphological analysis of microglia, z-stacks of 40-50 µm were acquired using the 63x objective (NA = 1.2), pinhole = 1 AU, voxel depth 0.5 µm, 0.18 µm/pixel. Two z-stacks were acquired per animal per region. Images were pre-processed using ImageJ functions for smoothing and background substraction (rolling ball). Images were then analysed using custom-written ImageJ plug-ins, as described previously ^5^. Briefly, binary images were obtained by application of an intensity threshold, which was determined in a 50% downscaled maximum projection of the original z-stack image using ImageJ‘s implemented threshold algorithm “MinError.” Individual cells were automatically traced in the z-stack by overlap. Particles smaller than 12,000 voxel were removed from the image. Surface area, volume, and convex hull were automatically reconstructed based on the size-filtered binary image. Skeleton parameters were obtained by Gauss-filtering (sigma = 0.5) of the size-filtered binary image and subsequent skeleton generation and analysis using the plugins by Arganda-Carreras and colleagues ^6^. Correct reconstruction of cells was visually verified and incorrectly traced cells (e.g. clustered or incomplete) corrected or excluded from analysis.

***Brain IBA1 / TMEM119 immunohistochemistry, image acquisition and analysis***

Coronal 60 µm free-floating brain sections were permeabilized and blocked in TBS-T 0.5% for 30 min, followed by heat-mediated antigen retrieval in citrate buffer (pH = 6) at 65°C for 20 min. After washing, sections were blocked in 10% NDS in TBS-T 0.5% for 3 h, followed by 48 h incubation at 4°C with primary antibodies against IBA1 and TMEM119, diluted in 5% NDS in TBS-T 0.25% (see Supplementary Table 1 for details). After washing, sections were stained with secondary antibodies for 4 h. Nuclei were stained with DAPI and washed sections mounted in Fluoromount-G®. Sections were imaged using a Leica TCS SP8 confocal microscope. For overview pictures, images were acquired with a 20x objective (NA = 0.75), pinhole = 1 AU, 0.28 µm/pixel). Two to three sections were imaged per animal for each region For co-localization analysis, images were acquired with a 40x objective (NA = 1.1), pinhole = 1 AU, 0.19 µm/pixel). Images were analysed using Fiji (ImageJ).

***Bone marrow TH immunohistochemistry, image acquisition and analysis***

For immunohistochemistry of bone marrow, tibias were post-fixed in 4% PFA for 3 days at 4°C, decalcified for 4 days at 4°C in EDTA solution (14% EDTA, 3% ammonium hydroxide, pH = 7.1), cryoprotected in 30% sucrose solution for 24 h at 4°C, embedded in TissueTek, and stored at -80°C. Bones were cut into 10 µm sections using a cryostat and stored at -80°C until further processing. To analyse SNS innervation of the bone marrow, tyrosine hydroxylase (TH) positive nerve endings were stained. Sections were washed in PBS, permeabilised in PBS-T 0.5% for 30 min, followed by washing in PBS and incubation in blocking solution (5% NGS in PBS-T 0.025%) for 1 - 2 h. Sections were then incubated with primary antibody against TH, diluted in antibody solution (2.5% NGS in PBS-T 0.025%), overnight at 4°C. On the next day, sections were washed, followed by 2 h incubation with secondary antibody. Nuclei were stained with DAPI and washed sections embedded in Fluoromount-G®. Stained bone marrow sections were imaged using a Leica TCS SP8 confocal microscope. Z-stacks of 10 µm were acquired using the 63x objective (numerical aperture (NA) 1.2), with an optical section thickness of 1 µm. Images were acquired as 8-bit, with 920 x 920 pixels. Next to TH and DAPI, a transmitted light picture was taken for identification of blood vessels. Five to seven images were acquired per animal. Images were analysed using Fiji (ImageJ 2.0.0). Maximum projections of z-stacks (TH channel) were used. Signal in the TH channel was thresholded to limit the analysis to TH-positive nerve endings. Within this threshold, TH signal intensity was measured as mean grey.

***Isolation of microglia for RNA-Sequencing***

Microglia (CD11b+ cells) were isolated from brain tissue of stress-naïve mice using magnetic cell separation. Briefly, male *Cnr1^+/+^* and *Cnr1^-/-^* mice (n = 3 per genotype) were deeply anaesthetised and perfused with ice-cold PBS. Brains were isolated and kept on ice in FACS buffer (HBSS + 2% FCS). After removing the cerebellum, brains were mechanically dissociated in glass douncers in 5 ml FACS buffer + 20 µg/ml DNase I (Roche). Homogenates were filtered through a 70 µm cell strainer, washed with FACS buffer and centrifuged (300 x g, 10 min, 4°C). For myelin removal, cells were resuspended in 40% Percoll and centrifuged (300 x g, 30 min, 4°C), after which myelin and supernatant were removed and pelleted cells washed in FACS buffer. Cells were counted and subjected to magnetic cell separation, using CD11b MicroBeads (MACS®, Miltenyi Biotec). In brief, cells were incubated with CD11b MicroBeads for 15 min at 4°C. After washing and centrifugation, cells were resuspended in 500 µl FACS buffer and transferred to MS columns placed in an OctoMACS separator. Columns were washed with FACS buffer to wash out unlabelled cells. To collect enriched CD11b+ cells, columns were removed from the magnet and cells flushed out into collection tubes. Cells were washed, centrifuged, and cell pellets snap-frozen in liquid nitrogen and stored at -80°C.

***RNA isolation, 3′ mRNA-Seq library preparation, and RNA-Seq***

Total RNA was isolated from enriched microglia using the Qiagen RNeasy Mini Kit according to the manufacturer’s instructions and eluted in 30 µl of RNAse-free water. Subsequent processing of RNA was performed at the NGS core facility of the University of Bonn. Quality control was performed using an Agilent TapeStation. Libraries for sequencing were prepared using the QuantSeq 3′ mRNA-Seq Library Prep Kit FWD for Illumina (Lexogen) as per manufacturer instructions. Reads were sequenced on an Illumina HiSeq 2500 V4 in high output mode. Approximately 10 million single-reads of 50 bp length were obtained per sample library. RNA-Seq data can be accessed under GSE152266.

***Gene expression analysis using Partek****®* ***Flow****®*

Analysis was performerd using the Lexogen QuantSeq pipeline implemented in Partek® Flow® Genomic Analysis Software, with small modifications. Briefly, trimmed reads were aligned to the mm10 reference index using STAR - 2.6.1d (deviation from default settings: filtering BySJout, max. read mapping 20, max. mismatches 99, mismatch mapped ratio 0.6, min. intron size 20, min. spliced alignment overhang 8, min. annotated spliced alignment overhang 1). Alignment rates were approximately 80-90% for all samples. Aligned reads were quantified using Partek E/M against mm10 - Ensembl Transcript release 99. Gene counts were normalised using TMM normalisation. To analyse overall sample similarity, normalised, log2-transformed counts were used for hierarchical clustering, principal component analysis (PCA), and t-distributed Stochastic Neighbor Embedding (tSNE). Differential gene expression analysis was performed using one-factorial ANOVA. A low-expression filter was applied to exclude genes with an average coverage of less than 10 and Limma-voom shrinkage of error term variance was applied. Multiple test correction was performed using FDR step-up ^7^. A summary report of the ANOVA is given in Supplementary Table 2. Differentially expressed (DE) genes were considered significant when reaching FDR < 0.1 and a fold change of > 1.5. For heat map visualisation of the top DE genes, results were filtered for nominal p-values < 0.01 and fold change of > 1.5.

**Supplementary figure legends**

**Supplementary Figure 1. Behavioural responses to standard CSDS in surviving *Dagla^fl/fl^*and *Dagla^-/-^*mice. (a)** Experimental timeline for standard CSDS experiments done in mice lacking DAGL
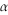
 - the enzyme synthesising the main endocannabinoid 2-AG. *Dagla^fl/fl^* littermates were used as wild type controls. **(b)** Similar to *Cnr1^-/-^* mice, *Dagla^-/-^*mice showed high mortality rates during exposure to standard CSDS. **(c - h)** Behavioural analysis of surviving mice after standard CSDS. **(c)** Social avoidance behaviour was analysed as interaction with a novel CD1 target mouse over a period of five minutes. In these experiments, no trial without CD1 target was performed (stress: F_(1,31)_ = 20.31, p < 0.0001; genotype: F_(1,31)_ = 8.38, p = 0.007). **(d)** Time spent in corner areas in the presence of a CD1 target (stress: F_(1,33)_ = 3.10, p = 0.088; genotype: F_(1,33)_ = 3.10, p = 0.088). **(e)** Anxiety in the open field test was measured as time spent in the centre area (no significant main effects)**. (f)** Anhedonia was measured in the sucrose preference test (no significant main effects). **(g)** Body weight change after 10 days of standard CSDS (no significant main effects). **(h)** Stress caused enlarged adrenal glands, measured 12 days after the last stress exposure (stress: F_(1,32)_ = 7.29, p = 0.011). Data was analysed by 2-way ANOVA, followed by Bonferroni *post-hoc* comparisons. For genotype effects (compared to *Dagla^fl/fl^* of the same group) * p < 0.05. For stress effects (compared to control of the same genotype) + p < 0.05.

**Supplementary Figure 2. Behavioural, endocrine, and immunological responses to standard CSDS in surviving *Cnr1^+/+^* and *Cnr1^-/-^* mice. (a)** Mice in cohort one were subjected to the social avoidance test after standard CSDS. First trial (no target): the cage located on one side of the arena is empty. Second trial (target):  cage containing an unfamiliar CD1 aggressor mouse. After all mice had completed the first trial, the empty cages were replaced by cages with CD1 mice and all mice tested in the second trial. Interaction with empty cage (genotype: F_(1,16)_ = 4.086, p = 0.0603). Social interaction (SI) ratio, calculated from the interaction time in the first and second trial (see Figure 1). A ratio > 1 (red line) is usually used to identify stress-resilient mice (stress: F_(1,16)_ = 46.1, p < 0.0001). Time spent in any of the four corners with no target (stress: F_(1,16)_ = 3.934, p = 0.0647; stress x genotype interaction: F_(1,16)_ = 4.022, p = 0.0621) or when the target is present (stress: F_(1,16)_ = 33.43, p < 0.0001). **(b)** Body weight change after standard CSDS, compared to day one (stress: F_(1,37)_ = 18.69, p = 0.0001). **(c)** Relative adrenal weight, measured approximately 24 h after the last stress exposure (stress: F_(1,16)_ = 4.844, p = 0.0428; genotype: F_(1,16)_ = 5.19, p = 0.0368). **(d - e)** In an independent cohort of mice, corticosterone (CORT) was measured after standard CSDS in blood plasma and fecal extracts using a CORT enzyme immunoassay (EIA). **(d)** Blood was collected approximately 24 h after the last stress exposure, at the beginning of the active phase (genotype: F_(1,18)_ = 3.885, p = 0.0643; stress x genotype interaction: F_(1,14)_ = 3.776, p = 0.0724). **(e)** In another cohort of mice, feces were collected from a 24 h period after standard CSDS. Steroids were extracted from fecal samples using ethanol extraction and used for CORT measurements (no significant main effects). **(f)** After standard CSDS, whole blood was collected by cardiac puncture and analysed by flow cytometry. Ly6C^hi^ monocyte frequency (stress: F_(1,15)_ = 5.81, p = 0.029). **(g)** Spleen weight (normalised to body weight) measured 24 h after the last stress exposure (stress: F_(1,16)_ =  13.34, p = 0.0021; genotype: F_(1,16)_ = 3.956, p = 0.0641). **(h - i)** Isolated splenocytes were cultured overnight and stimulated with LPS or vehicle (PBS). Afterwards, cells were harvested for flow cytometric analysis and supernatants collected for cytokine measurements. **(h)** Ly6C^hi^ monocyte frequency in unstimulated splenocytes (stress: F_(1,15)_ = 24.9, p = 0.0002). **(i)** IL-6 released from splenocytes stimulated with 1 µg/ml LPS (stress: F_(1,14)_ = 14.12, p = 0.002). Similar results were also observed for TNFa and IL-1b (data not shown). Data was analysed by 2-way ANOVA, followed by Bonferroni *post-hoc* comparisons. For genotype effects (compared to *Cnr1*^+/+^ of the same group) * p < 0.05, ** p < 0.01, *** p < 0.001. For stress effects (compared to control of the same genotype) + p < 0.05, ++ p < 0.01, +++ p < 0.001.

**Supplementary Figure 3. Stress susceptibility in the social avoidance test does not correlate with other stress-related behavioural changes. (a - d)** In cohort two of standard CSDS (5 min daily defeat), behavioural analysis of *Cnr1*^+/+^ mice showed that separation of stressed mice into susceptible and resilient groups in the social avoidance test does not segregate mice in other behavioural domains. **(a)** Social avoidance test: calculation of the SI ratio was used to allocate mice into susceptible (SI ratio < 1) and resilient (SI ratio > 1) groups. When a CD1 target was present, this separation clearly showed differences between resilient and susceptible mice in interaction time with the CD1 target (F = 13.47, p = 0.001) and time spent in the corners (F = 10.71, p = 0.002). **(b)** In contrast, susceptible and resilient mice did not show differences in anxiety in the open field, measured as time spent in the centre (F = 3.35, p = 0.07) and frequency to enter the centre (F = 3.43, p = 0.07). **(c)** In the sucrose preference, no significant difference was observed between resilient and susceptible mice (F = 2.75, p = 0.11). It even seemed like resilient mice showed lower preferences, indicative of stronger anhedonia. Data was analysed by 1way ANOVA followed by Bonferroni *post-hoc* comparisons, * p < 0.05. **(d)** Plasma IL-6 measured one hour after the first stress exposure was not correlated with the degree of later social avoidance behaviour in stressed *Cnr1*^+/+^ mice (R^2^ = 0.0142, p = 0.743). **(e)** Behavioural analysis after mild CSDS further supports that stress susceptibility in the social avoidance test is not sufficient to generally predict stress-related behaviour. The SI ratio of mice only correlated with parameters measured during the social avoidance test (time in corners: R^2^ = 0.36, p < 0.0001), but was not correlated with other behavioural readouts, such as open field anxiety (R^2^ = 0.009), p = 0.50), sucrose preference (R^2^ = 0.001), p = 0.80), or nest building behaviour (R^2^ = 0.001), p = 0.83). Data was analysed by Pearson correlation.

**Supplementary Figure 4. Effects of mild CSDS on brain IBA1 and ICAM-1 immunoreactivity.** Coronal brain sections of 60 µm were stained for IBA1 (red) and ICAM-1 (cyan), nuclei were stained with DAPI (blue). Sections were imaged using confocal microscopy. **(a)** Left panels: representative images of the dentate gyrus (DG) of the hippocampus, acquired with a 20x objective. Z-stacks of 9 µm were taken with a step size of 3 µm. Maximum projections are shown. Scale bar = 200 µm. Right panel: higher magnification images of the inferior molecular layer (indicated by white boxes in overview) were acquired using the 63x objective. Z-stacks of 40 – 50 µm were acquired with a step size of 0.5 µm. Maximum projections of 5 µm depth are shown. Scale bar = 25 µm. **(b)** Number of microglia (IBA1+ cells) in the DG (no significant main effects). **(c)** To analyse ICAM-1 expression by microglia, ICAM-1 mean grey within IBA1+ area was quantified in the molecular layer of the DG (no significant main effects). **(d)** Representative image of IBA1/ICAM-1 immunoreactivity in the CA1 region of the hippocampus, amygdala (AMY), and medial prefrontal cortex (mPFC). Images of a stressed *Cnr1^+/+^* mouse are shown exemplarily. Scale bar = 50 µm. **(e)** Quantification of IBA1+ area in the CA1 region (no significant main effects); AMY (stress x genotype interaction: F_(1,20)_ = 9.37, p = 0.006); mPFC (no significant main effects). **(f)** Quantification of ICAM-1+ area in the CA1 region (stress: F_(1,19)_ = 5.39, p = 0.032; stress x genotype interaction: F_(1,19)_ = 3.21, p = 0.089); AMY (stress: F_(1,17)_ = 3.27, p = 0.088); mPFC (stress: F_(1,18)_ = 4.17, p = 0.056; stress x genotype interaction: F_(1,18)_ = 3.72, p = 0.070). Data was analysed by 2-way ANOVA, followed by Bonferroni *post-hoc* comparisons. For genotype effects (compared to *Cnr1*^+/+^ of the same group) * p < 0.05. For stress effects (compared to control of the same genotype) + p < 0.05.

**Supplementary Figure 5. Mild CSDS-induced changes in IBA1+ area are mediated by brain resident TMEM119+ microglia.** Coronal brain sections of 60 µm were stained with antibodies against IBA1 (red) and TMEM119 (green), nuclei were stained with DAPI (blue). Sections were imaged using confocal microscopy. **(a)** Upper panels: representative image of the hippocampus of a *Cnr1*^+/+^ control mouse, acquired with a 20x objective, a single z-plane is shown. Scale bar = 200 µm. Bottom panel: higher magnification images of region of interest (indicated by white boxes in panel above). Arrows indicate IBA1+ TMEM119- cells, representing perivascular / meningeal macrophages. Mostly, those cells were found in proximity to blood vessels or meningeal regions and also displayed a round or rod-shaped morphology, rather different from the morphology of typical microglia. Arrow heads indicate IBA1+, TMEM119^low^ cells with ramified morphology. Only a very small number of IBA1+ TMEM119^low^ cells with ramified morphology were found in the regions analysed. An interesting observation was that TMEM119 immunoreactivity was generally weaker in the granule cell layer and the subgranular zone of the DG, a region associated with neurogenesis. IBA1+ cells in this area did have a faint TMEM119 signal, however it was much weaker compared to other hippocampal regions. Scale bar = 50 µm. **(b)** Quantification of TMEM119+ area in the DG in 20x overview images (stress x genotype interaction: F_(1,20)_ = 3.06, p = 0.096). Data was analysed by 2-way ANOVA, followed by Bonferroni *post-hoc* comparisons. **(c)** Pearson correlation analysis of IBA1+ vs. TMEM119+ area in the DG (R^2^ = 0.87, p < 0.0001).

**Supplementary Figure 6. Effects of mild CSDS on hippocampal microglial morphology.** Microglia morphology was analysed from 40 - 50 µm z-stacks acquired with 63x magnification, using an ImageJ-based analysis tool. **(a)** Additional morphological parameters of microglia within the molecular layer of the dentate gyrus. Cell surface area (stress: F_(1,266)_ = 12.58, p = 0.0005, stress x genotype interaction: F_(1,266)_ = 5.93, p = 0.016). Spanned volume of the convex hull (stress: F_(1,266)_ = 17.11, p < 0.0001, stress x genotype interaction: F_(1,266)_ = 3.61, p = 0.058). Number of junctions (stress: F_(1,269)_ = 8.30, p = 0.004). Total tree length, i.e. length of all processes (stress: F_(1,269)_ = 11.63, p = 0.0007). Average branch length (no significant main effects). **(b)** Microglia morphology within the stratum radiatum of the CA1 region. Although the directions of effects were similar to those observed in the DG, there were no significant differences for any morphological parameter analysed. Only IBA1 average intensity was affected by both stress and genotype (stress: F_(1,187)_ = 3.47, p = 0.006, genotype: F_(1,187)_ = 7.90, p < 0.0001, stress x genotype interaction: F_(1,187)_ = 2.63, p = 0.017). Data was analysed by 2-way ANOVA, followed by Bonferroni *post-hoc* comparisons. For genotype effects (compared to *Cnr1*^+/+^ of the same group) * p < 0.05. For stress effects (compared to control of the same genotype) + p < 0.05.

**Supplementary Figure 7. Transcriptome analysis of microglia (enriched CD11b+ cells) isolated from *Cnr1^+/+^* and *Cnr1^-/-^* mice.** Microglia were isolated from brain tissue of stress-naïve male mice (n = 3 per genotype) using magnetic cell separation with CD11b MicroBeads. Gene expression of isolated microglia was analysed using 3’ mRNA sequencing. **(a)** Summary of raw read counts and post-alignment quality control. **(b-d)** Analysis of overall sample similarity based on TMM-normalised, log2-transformed gene counts did not reveal a clear segregation of samples by genotype in **(b)** hierarchical clustering, **(c)** principal component analysis (PCA), or **(d)** t‑distributed Stochastic Neighbor Embedding (tSNE).

**Supplementary references:**

1. Zimmer, A., Zimmer, A. M., Hohmann, A. G., Herkenham, M. & Bonner, T. I. Increased mortality , hypoactivity , and hypoalgesia in cannabinoid CB1 receptor knockout mice. *Proc. Natl. Acad. Sci.* **96**, 5780–5785 (1999).

2. Jenniches, I. *et al.* Anxiety, Stress, and Fear Response in Mice with Reduced Endocannabinoid Levels. *Biol. Psychiatry* **79**, 858–868 (2016).

3. Bult, A. & Lynch, C. B. Nesting and fitness: lifetime reproductive success in house mice bidirectionally selected for thermoregulatory nest-building behavior. *Behav. Genet.* **27**, 231–240 (1997).

4. Moretti, P., Bouwknecht, J. A., Teague, R., Paylor, R. & Zoghbi, H. Y. Abnormalities of social interactions and home-cage behavior in a mouse model of Rett syndrome. *Hum. Mol. Genet.* **14**, 205–220 (2005).

5. Plescher, M. *et al.* Plaque-dependent morphological and electrophysiological heterogeneity of microglia in an Alzheimer’s disease mouse model. *Glia* **66**, 1464–1480 (2018).

6. Arganda-Carreras, I., Fernandez-Gonzalez, R., Munoz-Barrutia, A. & Ortiz-De-Solorzano, C. 3D reconstruction of histological sections: Application to mammary gland tissue. *Microsc. Res. Tech.* **73**, 1019–1029 (2010).

7. Benjamini, Y. & Hochberg, Y. Controlling the False Discovery Rate: A Practical and Powerful Approach to Multiple Testing. *J. R. Stat. Soc. Ser. B* **57**, 289–300 (1995).
