# Supplementary figures and images for: Cannabinoid receptor 1 signalling modulates stress susceptibility and microglial responses to chronic social defeat stress
Source: Transl Psychiatry. 2021 Mar 15;11:164. doi: 10.1038/s41398-021-01283-0 (PMC7961142; doi:10.1038/s41398-021-01283-0)

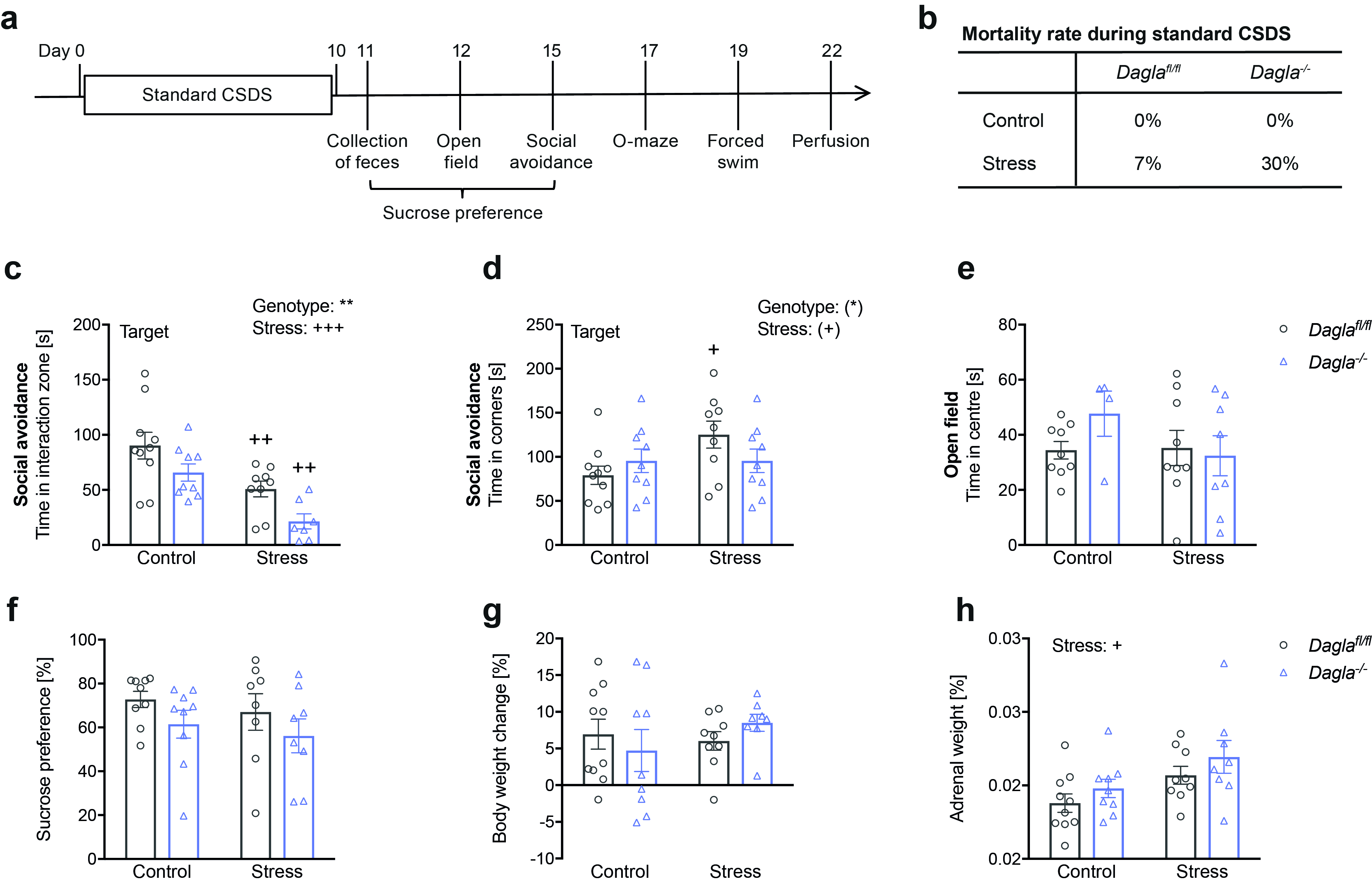

Supplement: Supplementary file 2 — Supplementary Figure 1 [file 41398_2021_1283_MOESM2_ESM.jpg]

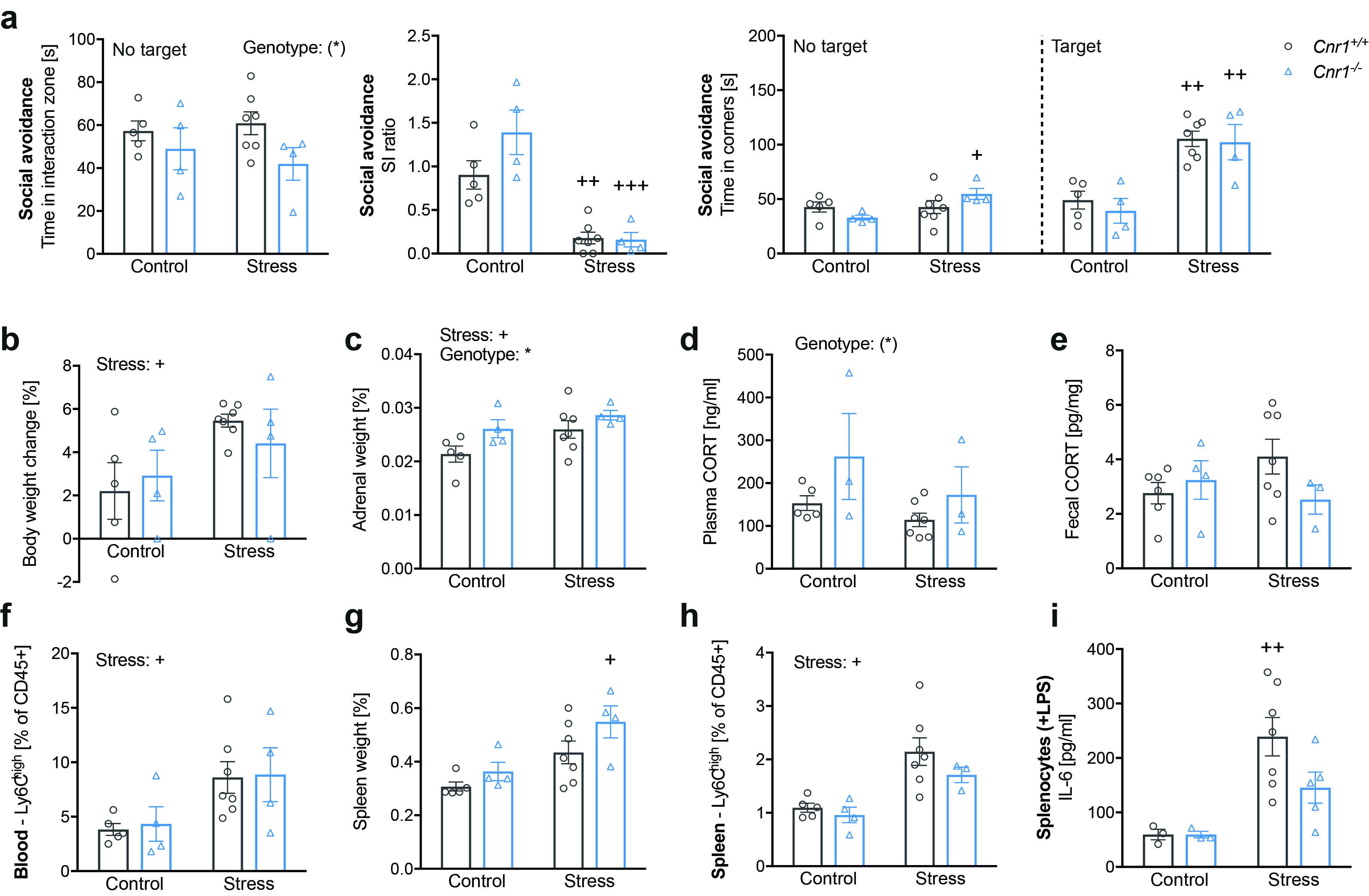

Supplement: Supplementary file 3 — Supplementary Figure 2 [file 41398_2021_1283_MOESM3_ESM.jpg]

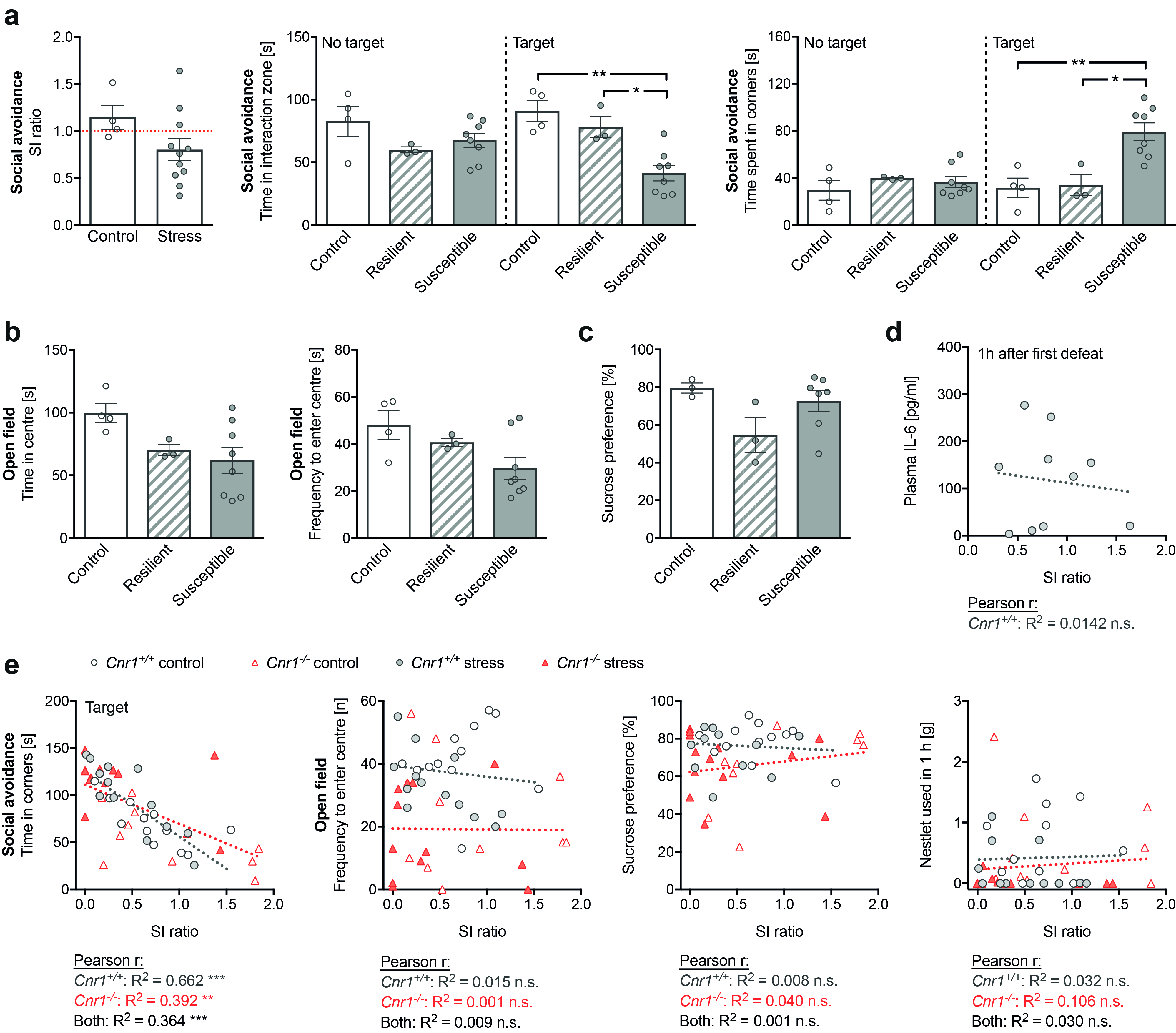

Supplement: Supplementary file 4 — Supplementary Figure 3 [file 41398_2021_1283_MOESM4_ESM.jpg]

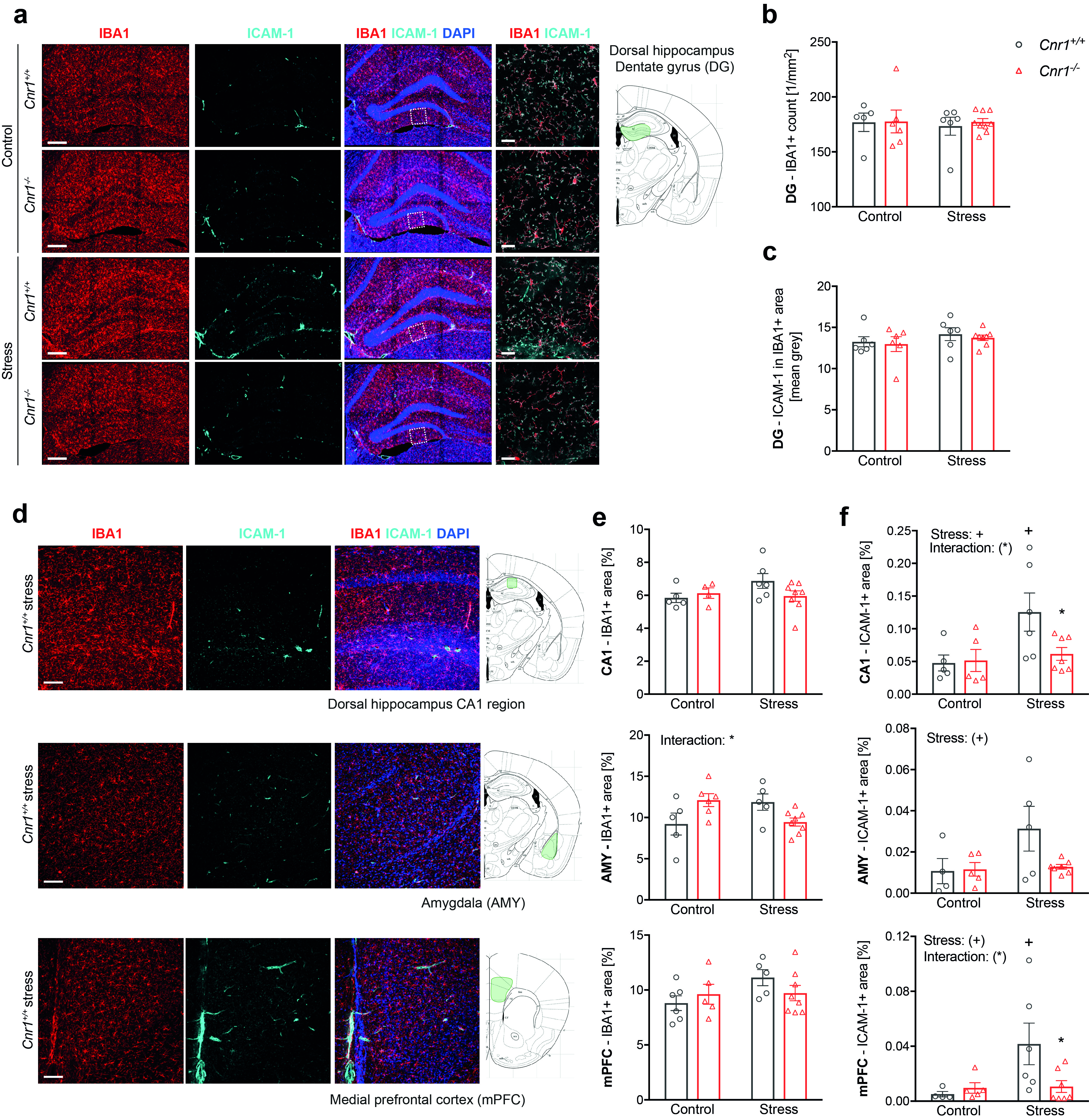

Supplement: Supplementary file 5 — Supplementary Figure 4 [file 41398_2021_1283_MOESM5_ESM.jpg]

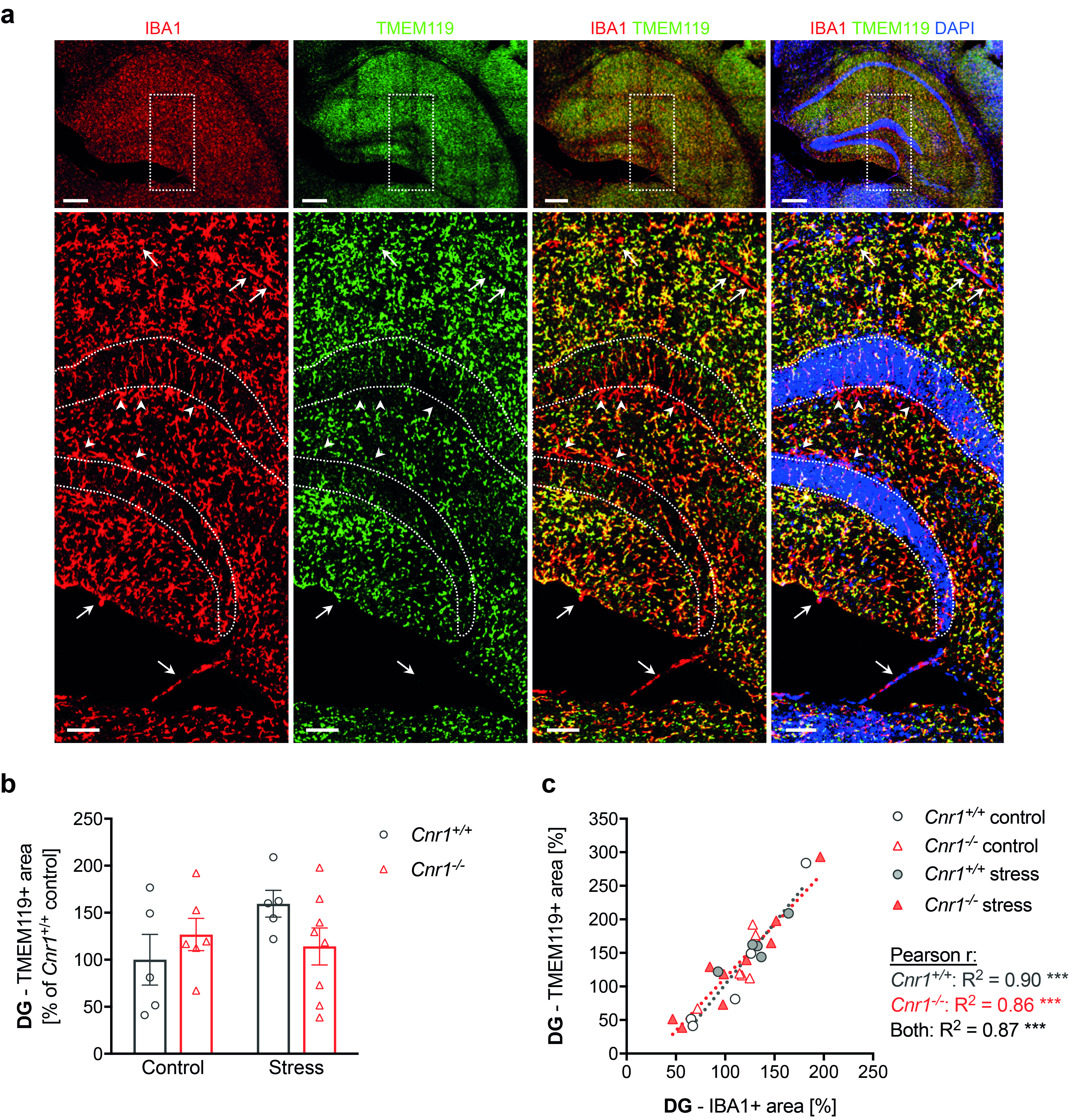

Supplement: Supplementary file 6 — Supplementary Figure 5 [file 41398_2021_1283_MOESM6_ESM.jpg]

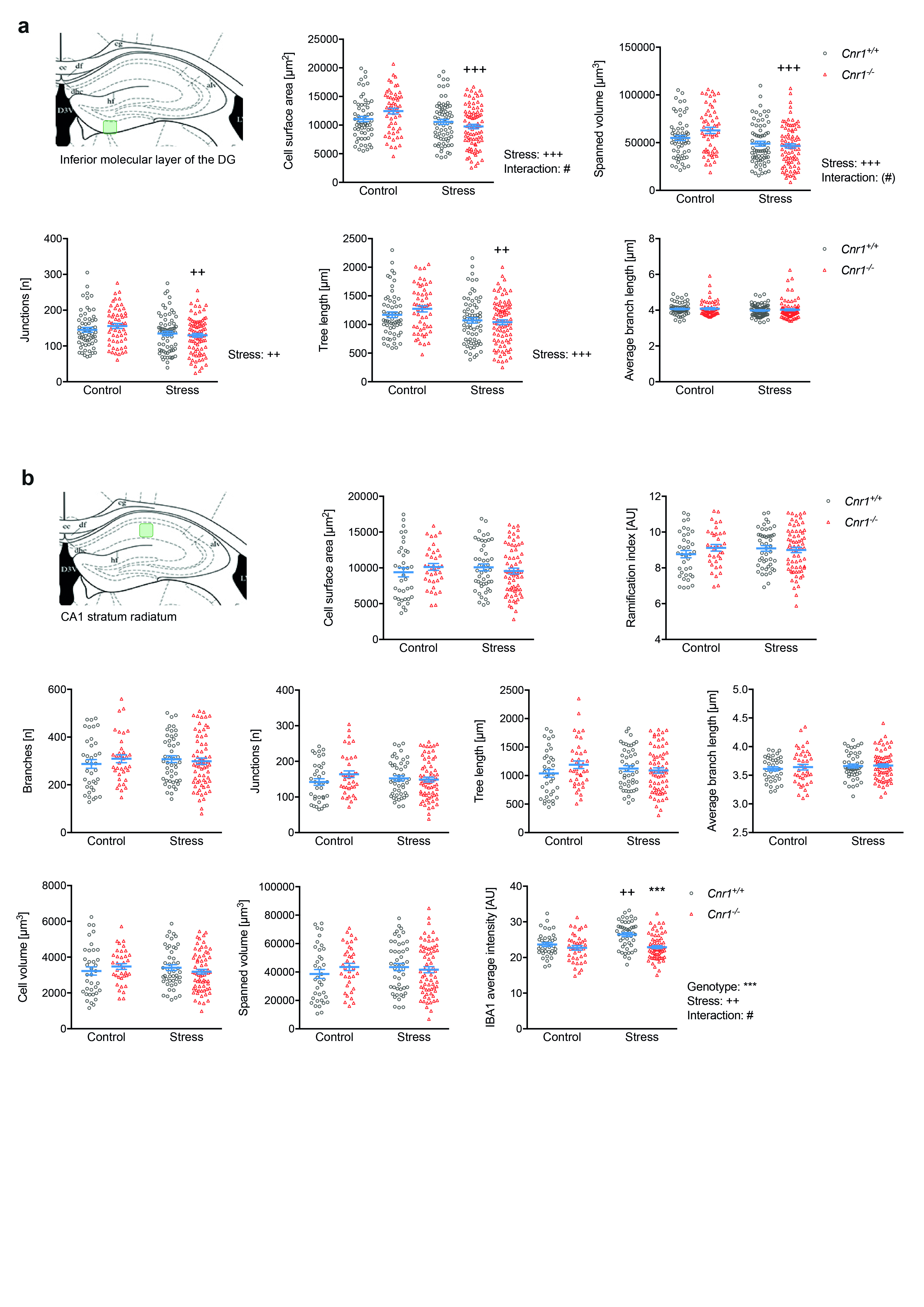

Supplement: Supplementary file 7 — Supplementary Figure 6 [file 41398_2021_1283_MOESM7_ESM.jpg]

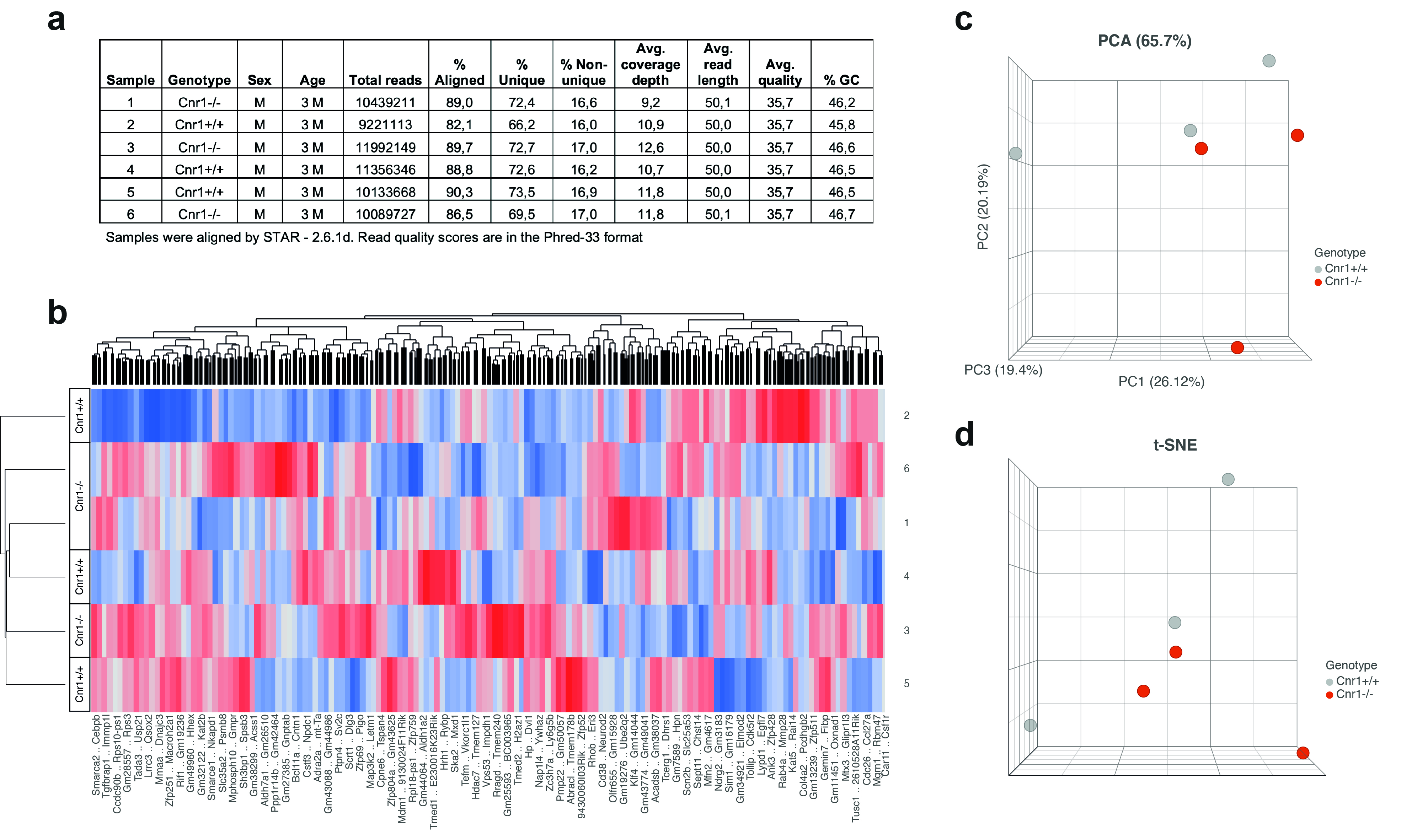

Supplement: Supplementary file 8 — Supplementary Figure 7 [file 41398_2021_1283_MOESM8_ESM.jpg]
