# Supplementary material for: Cannabinoid receptor 1 signalling modulates stress susceptibility and microglial responses to chronic social defeat stress
Source: Transl Psychiatry. 2021 Mar 15;11:164. doi: 10.1038/s41398-021-01283-0 (PMC7961142; doi:10.1038/s41398-021-01283-0)
Supplement: Supplementary file 9 — Supplementary Table 1 [file 41398_2021_1283_MOESM9_ESM.pdf]

Supplementary Table 1. Antibody information

Application	Antigen (all anti-mouse)	Conjugate	Host species	Clone	Dilution	Company	Catalog #
Flow cytometry	CD115 (CSF-1R)	PerCP/Cy5.5	Rat	AFS98	1:100	BioLegend	135526
	NK1.1	Biotin	Rat	PK136	1:200	BioLegend	108704
	TER-119	Biotin	Rat	TER-119	1:200	BioLegend	116204
	Ly-6G	Biotin	Rat	1A8	1:200	BioLegend	127604
	Ly-6G	PE/Cy7	Rat	1A8	1:200	BD	560601
	CD3	Biotin	Rat	17A2	1:200	BioLegend	100244
	CD45	Brilliant Violet 510	Rat	30-F11	1:200	BioLegend	103137
	CD11b	PE/Cy7	Rat	M1/70	1:200	BioLegend	101216
	CD11b	FITC	Rat	M1/70	1:200	BD	553310
	Ly-6C	APC	Rat	HK1.4	1:200	BioLegend	128016
	CD19	APC/Cy7	Rat	1D3	1:200	BD	557655
	CCR2 (CD192)	Brilliant Violet 421	Rat	SA203G11	1:200	BioLegend	150605
	MERTK (Mer)	PE	Rat	2B10C42	1:400	BioLegend	151505
	MHC class II (I-A/I-E)	PerCP/Cy5.5	Rat	M5/114.15.2	1:200	BioLegend	107625
	CD11c	FITC	Hamster	N418	1:200	BioLegend	117306
	CD40	PE	Rat	1C10	1:200	eBioscience	12-0401
	Streptavidin	APC/Cy7			1:200	BioLegend	405208
Immunohistochemistry	IBA1	unconjugated	Rabbit	Polyclonal	1:1000	Wako	019-19741
	IBA1	unconjugated	Goat	Polyclonal	1:1000	abcam	ab5076
	ICAM-1/CD54	unconjugated	Goat	Polyclonal	1:1000	R&D systems	AF796
	TH	unconjugated	Rabbit	Polyclonal	1:1000	abcam	ab112
	TMEM119	unconjugated	Rabbit	43918	1:1000 (0.614 µg/ml)	abcam	ab234501
	CD45	unconjugated	Rat	IBL-3/16	1:500	abcam	ab23910
	Anti-rat IgG	Alexa Fluor 488	Donkey		1:1000	Invitrogen	A21208
	Anti-rabbit IgG	Alexa Fluor 594	Donkey		1:1000	Invitrogen	A21207
	Anti-goat IgG	Alexa Fluor 647	Donkey		1:1000	Invitrogen	A21447
