# Supplementary material for: Cannabinoid receptor 1 signalling modulates stress susceptibility and microglial responses to chronic social defeat stress
Source: Transl Psychiatry. 2021 Mar 15;11:164. doi: 10.1038/s41398-021-01283-0 (PMC7961142; doi:10.1038/s41398-021-01283-0)
Supplement: Supplementary file 10 — Supplementary Table 2 [file 41398_2021_1283_MOESM10_ESM.pdf]

Supplementary Table 2. ANOVA summary report: Differentially expressed (DE) genes between Cnr1+/+ vs. Cnr1-/- microglia; DE genes with a p value < 0.05 (uncorrected) are listed.

Gene symbol	Total counts	P-value (Cnr1-/- vs, Cnr1+/+)	FDR step up (Cnr1-/- vs, Cnr1+/+)	Fold change (Cnr1-/- vs, Cnr1+/+)	LSMean(Cnr1-/-) (Cnr1-/- vs, Cnr1+/+)	LSMean(Cnr1+/+) (Cnr1-/- vs, Cnr1+/+)
Fkbp5	362.58	3.03E-07	0.002	2.34	82.10	35.14
Arsb	444.04	9.84E-07	0.003	2.41	99.89	41.39
Hist1h2ap	570.48	3.07E-06	0.007	2.27	126.35	55.59
Gm11353	155.38	5.81E-06	0.009	-2.56	14.11	36.14
Ttc28	612.50	1.22E-05	0.016	1.77	127.86	72.35
Ogfrl1	1083.54	3.15E-05	0.030	1.49	216.31	144.85
Ddit4	622.23	3.24E-05	0.030	2.76	136.29	49.31
Adrb1	710.40	9.79E-05	0.080	1.59	143.72	90.37
Gm21860	98.65	1.88E-04	0.136	-3.17	6.85	21.69
Rpl18-ps2	138.25	2.24E-04	0.147	1.97	30.06	15.28
Gm5905	105.38	4.76E-04	0.283	1.94	22.60	11.66
Sult1a1	405.25	7.24E-04	0.315	1.48	80.16	54.00
G530011O06Rik	126.47	7.39E-04	0.315	-1.92	13.61	26.15
Gm49839	110.04	7.61E-04	0.315	2.55	23.74	9.32
Gm15501	233.96	7.63E-04	0.315	1.67	47.84	28.61
Ltf	159.24	7.72E-04	0.315	4.06	28.18	6.94
Klf13	340.78	8.54E-04	0.328	1.47	67.59	45.86
mt-Atp8	678.56	9.05E-04	0.329	1.45	132.57	91.14
Tagln2	157.62	1.04E-03	0.354	-1.67	19.56	32.65
Rtcb	235.47	1.08E-03	0.354	1.52	47.29	31.04
Ppp1r14b	174.51	1.27E-03	0.395	1.59	35.47	22.32
Otulin	99.92	1.41E-03	0.416	1.78	21.19	11.91
Nfkbia	1124.99	1.46E-03	0.416	1.47	219.27	149.55
Abcc1	71.34	1.56E-03	0.424	-1.90	7.93	15.09
Cd14	709.12	1.63E-03	0.425	1.37	136.51	99.63
Ccnd3	238.32	1.73E-03	0.434	1.49	47.37	31.81
Abcg1	305.05	1.86E-03	0.434	1.47	60.14	40.99
Fbxw2	130.34	1.89E-03	0.434	1.62	26.89	16.58
Cd74	239.54	1.92E-03	0.434	-1.49	32.05	47.70
Rcbtb2	455.14	2.23E-03	0.479	1.40	88.24	63.23
Dctn3	91.97	2.27E-03	0.479	1.73	19.41	11.21
Lrrc3	597.83	2.38E-03	0.486	1.41	115.53	82.04
Gid8	80.52	2.54E-03	0.503	1.77	16.95	9.58
Lta4h	68.80	3.05E-03	0.539	1.77	14.60	8.24
Ptprj	236.51	3.07E-03	0.539	1.49	46.90	31.51
Ucp2	533.50	3.08E-03	0.539	1.43	102.72	71.99
Ncf1	1657.24	3.10E-03	0.539	1.29	310.93	240.18
Isca1	126.83	3.23E-03	0.539	1.60	26.00	16.25
Lifr	303.31	3.23E-03	0.539	1.41	59.03	41.74
Med22	183.05	3.32E-03	0.539	1.50	36.43	24.24
Gm3739	215.59	3.59E-03	0.539	1.50	43.00	28.62
S100a8	2043.30	3.63E-03	0.539	2.78	306.48	110.31
Tmem248	147.15	3.68E-03	0.539	1.53	29.50	19.30
AU020206	337.70	3.71E-03	0.539	1.40	65.51	46.88
Slc35a4	86.25	3.71E-03	0.539	-1.69	10.64	18.01
Rpl5	106.25	3.92E-03	0.553	1.73	21.56	12.45
Cox6a1	499.78	3.98E-03	0.553	1.34	95.43	71.22
Lemd3	83.40	4.14E-03	0.564	1.70	17.41	10.22
Psme2	130.34	4.33E-03	0.571	2.16	26.35	12.18
Lrrc17	3758.49	4.40E-03	0.571	1.38	712.06	516.39
Anapc16	220.34	4.46E-03	0.571	1.43	43.07	30.18
Ccl6	399.22	4.64E-03	0.573	1.36	76.56	56.48
Prr14l	74.65	4.64E-03	0.573	1.75	15.63	8.95
mt-Nd4l	318.70	4.80E-03	0.581	1.43	61.37	42.82
Tsc22d3	244.00	5.08E-03	0.581	1.49	48.15	32.26
Prkcb	400.60	5.23E-03	0.581	1.36	76.85	56.59
H2-Aa	241.58	5.27E-03	0.581	-1.40	33.47	46.87
Dab2	178.50	5.27E-03	0.581	-1.49	23.83	35.49
Amotl1	99.51	5.36E-03	0.581	-1.60	12.70	20.33

Paox	110.79	5.48E-03	0.581	1.65	22.29	13.47
Gpr157	83.25	5.51E-03	0.581	1.66	17.06	10.25
Elf4	112.61	5.52E-03	0.581	1.61	23.07	14.35
Spint1	233.04	5.89E-03	0.611	1.47	45.83	31.24
Slc12a2	254.35	6.25E-03	0.638	-1.43	34.82	49.73
H2-Eb1	254.36	6.66E-03	0.660	-1.39	35.23	48.87
Pnpla2	171.73	6.71E-03	0.660	1.45	33.88	23.35
Heatr5a	284.28	6.77E-03	0.660	1.42	55.30	39.03
Auts2	150.73	7.14E-03	0.668	-1.59	18.76	29.76
Prdx6	171.88	7.34E-03	0.668	1.50	33.77	22.48
Sall3	732.71	7.50E-03	0.668	1.30	137.86	105.74
Cacul1	164.97	7.60E-03	0.668	1.45	32.56	22.47
Itpripl2	201.90	7.70E-03	0.668	1.47	39.74	27.01
Ccnd2	87.40	7.79E-03	0.668	-1.68	10.65	17.85
Gm8129	149.93	7.93E-03	0.668	-1.55	19.38	29.95
Slc25a5	355.89	8.17E-03	0.668	1.34	67.82	50.50
Gm10925	10803.40	8.23E-03	0.668	1.25	1991.09	1594.75
Mrto4	63.87	8.38E-03	0.668	-1.68	7.78	13.10
Il17ra	905.84	8.38E-03	0.668	1.27	168.58	132.71
Slco4a1	193.97	8.42E-03	0.668	1.43	37.95	26.60
9530056E24Rik	71.16	8.51E-03	0.668	1.65	14.60	8.87
mt-Nd3	3871.30	8.61E-03	0.668	1.26	716.19	567.85
Per1	85.70	8.61E-03	0.668	1.71	17.59	10.31
Irf5	644.18	8.71E-03	0.668	1.30	121.30	93.26
mt-Atp6	8914.33	8.71E-03	0.668	1.25	1642.19	1316.48
Sumo2	171.04	8.73E-03	0.668	1.44	33.62	23.39
Klhl6	165.32	8.79E-03	0.668	1.55	32.70	21.15
Golm1	1952.02	9.09E-03	0.683	1.24	360.46	289.69
Arhgdia	347.54	9.51E-03	0.694	1.34	66.13	49.33
Gm10443	490.76	9.61E-03	0.694	1.29	92.14	71.26
Hbb-bs	906.57	9.75E-03	0.694	2.81	122.48	43.57
mt-Co2	14245.79	9.83E-03	0.694	1.24	2618.70	2109.60
Gcnt2	81.44	9.92E-03	0.694	1.59	16.45	10.35
mt-Cytb	14528.31	9.94E-03	0.694	1.25	2672.42	2144.46
Tmem176a	322.87	1.00E-02	0.694	1.33	61.33	46.08
Calhm2	157.29	1.01E-02	0.694	1.48	30.92	20.96
Ppan	70.70	1.02E-02	0.694	1.62	14.42	8.91
Timm9	112.73	1.03E-02	0.694	1.55	22.52	14.55
Tmem176b	1000.92	1.06E-02	0.696	1.27	185.71	146.60
Cables1	289.23	1.06E-02	0.696	1.40	55.38	39.45
Rbm26	737.96	1.07E-02	0.696	1.28	137.66	107.66
Pnpo	65.23	1.08E-02	0.696	1.64	13.23	8.08
Gnaq	375.36	1.10E-02	0.696	1.31	71.02	54.14
Ubr1	146.66	1.11E-02	0.696	1.56	29.15	18.64
R3hdm4	171.77	1.11E-02	0.696	1.43	33.42	23.38
Rnf169	299.63	1.12E-02	0.696	1.34	56.98	42.52
Cd34	460.15	1.13E-02	0.697	1.29	86.37	66.73
Pald1	241.58	1.15E-02	0.699	1.36	46.22	34.06
Sorl1	213.23	1.16E-02	0.700	1.37	41.16	30.01
Api5	139.75	1.22E-02	0.726	1.49	27.31	18.32
Plp1	1239.48	1.23E-02	0.726	-1.32	175.80	231.26
Dynlt1f	102.10	1.24E-02	0.726	-1.58	12.75	20.18
Cbfa2t3	289.84	1.24E-02	0.726	1.36	55.56	40.99
Chchd7	79.32	1.28E-02	0.738	1.57	16.09	10.22
Rhob	1811.55	1.31E-02	0.738	1.25	333.99	266.97
Arrb1	306.54	1.33E-02	0.738	1.33	58.27	43.87
Lrrc8d	137.60	1.34E-02	0.738	1.45	27.16	18.76
Gm12543	82.36	1.35E-02	0.738	-1.60	10.46	16.74
Rpl17	106.05	1.36E-02	0.738	-1.53	13.65	20.88
Hspa8	1299.37	1.38E-02	0.738	-1.26	190.91	240.40
Bc1	1476.86	1.39E-02	0.738	1.35	275.33	203.40
Dusp16	104.26	1.39E-02	0.738	1.61	20.93	12.96
Eif2ak1	120.60	1.40E-02	0.738	1.47	23.92	16.27
Gm26532	69.27	1.41E-02	0.738	-1.56	8.95	13.99

mt-Nd1	16429.71	1.41E-02	0.738	1.23	3003.72	2448.24
Cpsf6	286.45	1.41E-02	0.738	1.38	54.94	39.92
Gm43813	87.14	1.43E-02	0.743	1.69	17.60	10.44
Ppfia4	1042.77	1.46E-02	0.749	1.26	192.58	152.55
Arhgdib	348.32	1.47E-02	0.749	1.32	65.94	49.91
H2-Ab1	287.38	1.48E-02	0.750	-1.31	41.29	54.17
Lactb	325.21	1.50E-02	0.750	1.31	61.26	46.80
H2-DMa	320.68	1.52E-02	0.750	1.30	60.52	46.45
Asxl1	155.97	1.54E-02	0.750	1.40	30.35	21.64
Sgk1	654.63	1.54E-02	0.750	1.29	122.07	94.71
Rnf11	163.31	1.55E-02	0.750	1.40	31.65	22.59
Med11	69.30	1.56E-02	0.750	1.61	14.03	8.72
S100a9	2385.96	1.57E-02	0.750	2.32	332.45	143.37
Hipk1	286.14	1.58E-02	0.750	1.35	54.57	40.56
Smim3	88.83	1.58E-02	0.750	1.51	17.81	11.79
Drap1	304.86	1.60E-02	0.750	1.32	57.73	43.77
Xpc	76.83	1.63E-02	0.750	1.64	15.53	9.48
Gm44694	171.59	1.66E-02	0.750	1.41	33.11	23.42
Tatdn1	4141.12	1.67E-02	0.750	1.36	770.09	566.40
Gm26740	69.70	1.68E-02	0.750	1.58	14.11	8.93
Elmo1	608.36	1.70E-02	0.750	1.26	112.97	89.66
Pias3	94.20	1.71E-02	0.750	1.55	18.78	12.11
Slc36a1	504.03	1.72E-02	0.750	1.27	93.90	74.22
Gdpd3	67.85	1.72E-02	0.750	1.58	13.67	8.68
Ccng2	191.90	1.72E-02	0.750	1.42	37.29	26.33
Smpd5	66.95	1.73E-02	0.750	1.56	13.55	8.70
Ythdf2	511.34	1.74E-02	0.750	1.30	95.92	73.94
Gm12411	180.28	1.74E-02	0.750	1.37	34.66	25.24
Nenf	75.98	1.74E-02	0.750	-1.55	9.85	15.23
Pnpla6	63.63	1.77E-02	0.750	1.61	12.88	8.01
Map4k2	87.52	1.77E-02	0.750	1.58	17.64	11.17
Dgkz	189.08	1.80E-02	0.757	1.36	36.23	26.63
Gbp7	392.12	1.84E-02	0.759	1.29	73.61	56.91
Fads2	74.80	1.86E-02	0.759	1.60	14.82	9.25
Slc1a3	884.24	1.87E-02	0.759	1.25	162.74	130.63
Dock11	129.49	1.89E-02	0.759	1.45	25.50	17.62
Nipsnap2	79.56	1.89E-02	0.759	1.54	15.86	10.31
Arhgap25	330.37	1.90E-02	0.759	1.29	62.01	48.03
Axl	75.40	1.92E-02	0.759	-1.65	8.85	14.61
Jade2	286.64	1.94E-02	0.759	1.30	53.75	41.25
Hnrnpk	1005.21	1.95E-02	0.759	1.23	184.58	149.91
Plbd2	173.29	1.95E-02	0.759	1.37	33.05	24.09
Gm4356	81.84	1.95E-02	0.759	1.51	16.29	10.76
6030458C11Rik	73.75	1.97E-02	0.759	1.52	14.76	9.72
Tnfaip8	298.12	1.98E-02	0.759	1.29	56.07	43.39
Txnip	826.40	2.00E-02	0.759	1.24	152.23	122.33
Gm9616	107.95	2.01E-02	0.759	1.57	21.45	13.64
Csnk1g1	79.79	2.05E-02	0.759	1.54	15.99	10.36
Map7d2	151.39	2.06E-02	0.759	1.53	28.55	18.66
Tmem184c	93.56	2.06E-02	0.759	1.49	18.59	12.50
Pip5k1c	154.66	2.06E-02	0.759	1.45	30.11	20.70
Ube2g1	64.50	2.07E-02	0.759	1.59	12.97	8.15
Raph1	73.16	2.08E-02	0.759	-1.57	9.36	14.73
Tnfsf13b	67.80	2.08E-02	0.759	1.58	13.57	8.60
Il6ra	1716.87	2.10E-02	0.759	1.22	314.02	257.58
2510009E07Rik	173.93	2.10E-02	0.759	1.37	33.33	24.37
Cass4	63.48	2.10E-02	0.759	-1.54	8.31	12.83
Tmem37	179.14	2.12E-02	0.759	1.37	34.39	25.14
Ly86	670.32	2.13E-02	0.759	1.25	123.78	98.81
1110059G10Rik	115.44	2.13E-02	0.759	1.46	22.69	15.57
Gatad2a	199.92	2.18E-02	0.759	1.34	38.11	28.34
Pbxip1	863.38	2.18E-02	0.759	1.23	158.66	129.05
Mid1	82.74	2.18E-02	0.759	-1.66	9.53	15.83
Vamp2	195.94	2.19E-02	0.759	1.34	37.14	27.78

Cttnbp2	86.62	2.19E-02	0.759	-1.60	10.61	16.95
mt-Nd4	29927.91	2.19E-02	0.759	1.23	5451.09	4439.79
Camp	349.31	2.22E-02	0.759	2.83	47.61	16.80
Psmc6	195.45	2.24E-02	0.759	-1.33	27.92	37.12
Ttc39b	134.31	2.26E-02	0.759	1.46	26.19	17.88
Ctu2	81.78	2.26E-02	0.759	1.50	16.25	10.86
Lcn2	176.55	2.28E-02	0.759	2.63	26.28	9.99
Slc35e1	90.45	2.29E-02	0.759	1.49	17.98	12.10
Gpi1	271.82	2.29E-02	0.759	1.30	51.05	39.28
Abcd1	243.92	2.30E-02	0.759	1.30	45.91	35.23
Myo1c	78.53	2.30E-02	0.759	1.62	15.59	9.65
U2af1	185.00	2.31E-02	0.759	1.35	35.32	26.18
Brpf1	234.10	2.33E-02	0.759	1.33	44.14	33.30
mt-Nd5	3380.24	2.34E-02	0.759	1.25	618.48	495.18
Usp38	70.17	2.35E-02	0.759	1.56	13.93	8.93
Dlgap4	408.77	2.38E-02	0.759	1.27	76.23	59.98
Sfswap	480.98	2.39E-02	0.759	1.26	89.39	70.86
Spns2	74.22	2.40E-02	0.759	1.54	14.87	9.66
Gan	77.25	2.41E-02	0.759	1.52	15.42	10.17
Tnfrsf11a	205.16	2.41E-02	0.759	1.36	39.27	28.94
Klc2	66.95	2.42E-02	0.759	1.51	13.40	8.89
Kif5b	698.17	2.44E-02	0.759	1.25	128.66	103.13
Kat6a	460.29	2.44E-02	0.759	1.27	85.40	67.36
Gtf2a2	161.01	2.45E-02	0.759	1.37	30.98	22.66
Mlxip	87.45	2.46E-02	0.759	1.50	17.40	11.56
Naxd	83.29	2.50E-02	0.763	1.49	16.55	11.14
Aph1a	238.71	2.51E-02	0.763	1.30	44.94	34.51
Mto1	71.17	2.52E-02	0.763	1.54	14.19	9.21
Kif5c	152.70	2.52E-02	0.763	1.45	29.43	20.35
Scarb1	119.61	2.55E-02	0.763	1.40	23.28	16.58
Ppp4c	66.65	2.58E-02	0.763	-1.53	8.73	13.34
Enpp4	74.46	2.58E-02	0.763	-1.49	9.91	14.77
Arhgef1	487.40	2.58E-02	0.763	1.26	90.50	71.78
Neurl1a	328.39	2.58E-02	0.763	1.29	61.17	47.56
Tbc1d9	268.51	2.63E-02	0.763	1.28	50.24	39.15
Nop2	103.13	2.63E-02	0.763	1.43	20.26	14.17
Plgrkt	285.27	2.67E-02	0.763	1.28	53.28	41.71
Wdfy3	283.67	2.69E-02	0.763	1.29	53.12	41.30
Agpat5	86.80	2.72E-02	0.763	-1.49	11.52	17.14
Ankrd13a	220.68	2.72E-02	0.763	1.32	41.90	31.70
Sort1	572.78	2.72E-02	0.763	1.25	105.93	84.61
Rundc1	65.56	2.74E-02	0.763	1.55	12.95	8.38
G6pdx	63.77	2.75E-02	0.763	-1.50	8.50	12.76
Pias1	221.58	2.76E-02	0.763	1.30	41.83	32.11
Tbc1d31	64.35	2.77E-02	0.763	1.51	12.81	8.47
Whamm	98.29	2.77E-02	0.763	1.43	19.34	13.50
Pck2	173.45	2.78E-02	0.763	1.36	33.20	24.34
Cept1	308.18	2.78E-02	0.763	1.28	57.56	44.83
Camk1d	264.19	2.78E-02	0.763	1.28	49.45	38.54
Ubb	561.86	2.79E-02	0.763	1.25	103.89	82.78
Rps12-ps4	331.81	2.80E-02	0.763	1.33	62.11	46.67
Arpc5	327.30	2.81E-02	0.763	1.33	61.22	46.18
Zfp652	393.58	2.82E-02	0.763	1.25	72.96	58.22
Zfp746	66.68	2.82E-02	0.763	1.50	13.25	8.86
Nrsn1	67.38	2.83E-02	0.763	1.56	13.39	8.59
Fcf1	245.66	2.84E-02	0.763	1.30	46.02	35.48
Igsf6	177.19	2.85E-02	0.763	1.34	33.79	25.23
Foxo1	156.65	2.86E-02	0.763	-1.37	21.94	29.97
U2af2	497.19	2.89E-02	0.766	1.26	91.88	73.09
mt-Nd6	117.44	2.90E-02	0.766	1.52	22.45	14.76
Ykt6	328.99	2.94E-02	0.766	1.26	61.12	48.50
Slc22a17	211.53	2.94E-02	0.766	1.35	40.42	29.94
mt-Nd2	14487.33	2.95E-02	0.766	1.21	2625.53	2168.58
Commd2	66.95	2.96E-02	0.766	1.60	13.24	8.30

Ripor1	64.80	2.98E-02	0.766	1.53	12.93	8.47
Gatad2b	483.74	2.98E-02	0.766	1.25	89.41	71.76
Zfhx3	2639.04	2.99E-02	0.766	1.20	479.14	398.12
Vhl	187.86	3.00E-02	0.766	1.34	35.83	26.79
Pdp2	122.12	3.00E-02	0.766	1.47	23.79	16.19
Hbb-bt	305.67	3.03E-02	0.769	2.87	38.17	13.29
Trp53bp2	63.42	3.06E-02	0.774	1.51	12.61	8.35
Pomp	727.01	3.07E-02	0.775	1.23	133.03	108.54
Parg	86.20	3.12E-02	0.776	-1.45	11.65	16.84
Syvn1	180.35	3.12E-02	0.776	1.33	34.25	25.70
Impa1	112.62	3.14E-02	0.776	-1.42	15.48	21.94
Hypk	175.96	3.14E-02	0.776	1.35	33.18	24.63
2510039O18Rik	268.48	3.14E-02	0.776	1.28	49.96	38.90
Gm48690	86.92	3.16E-02	0.776	1.47	16.93	11.50
Rpl22l1	174.13	3.16E-02	0.776	1.33	33.07	24.78
Pbx3	73.96	3.19E-02	0.777	1.52	14.58	9.62
9330162G02Rik	195.03	3.19E-02	0.777	-1.32	27.68	36.65
Mbtd1	345.85	3.21E-02	0.777	1.25	64.20	51.20
Rpl38-ps2	359.60	3.25E-02	0.777	1.28	66.89	52.43
Pigt	117.52	3.25E-02	0.777	1.44	22.51	15.63
Eya3	64.28	3.27E-02	0.777	1.55	12.69	8.20
Nod1	68.13	3.29E-02	0.777	-1.57	8.52	13.37
Rpl18-ps1	89.20	3.31E-02	0.777	-1.49	11.71	17.41
Nt5c	84.75	3.32E-02	0.777	1.48	16.68	11.26
Hba-a1	559.36	3.34E-02	0.777	2.57	68.63	26.66
Frrs1	370.68	3.35E-02	0.777	1.25	68.46	54.60
Cebpz	834.40	3.36E-02	0.777	1.22	152.74	124.84
mt-Th	201.46	3.39E-02	0.777	1.30	37.92	29.24
Parvb	62.88	3.40E-02	0.777	-1.51	8.23	12.44
Rpl37rt	2157.65	3.41E-02	0.777	1.19	390.66	327.03
Csnk1e	1386.87	3.43E-02	0.777	1.19	251.45	210.73
Gtpbp2	261.51	3.43E-02	0.777	1.30	49.03	37.79
Zfp942	71.35	3.44E-02	0.777	1.51	14.06	9.31
Hba-a2	1379.56	3.46E-02	0.777	2.23	174.51	78.31
Gm12481	153.55	3.48E-02	0.777	1.41	29.20	20.72
Slc25a36	203.75	3.49E-02	0.777	1.30	38.36	29.48
Tef	391.13	3.49E-02	0.777	-1.25	57.51	71.94
Pacsin2	267.93	3.50E-02	0.777	1.28	50.08	39.01
Retnlg	131.10	3.51E-02	0.777	2.32	19.67	8.46
6720427I07Rik	65.15	3.52E-02	0.777	1.53	12.73	8.30
Rassf2	800.95	3.56E-02	0.777	1.21	146.11	120.73
Fam102a	64.90	3.56E-02	0.777	1.47	12.78	8.68
Gatm	170.46	3.58E-02	0.777	1.33	32.28	24.35
Mtus1	811.27	3.60E-02	0.777	1.22	148.39	121.62
Pmepa1	3109.03	3.61E-02	0.777	1.18	561.34	473.76
Mafg	874.98	3.63E-02	0.777	1.20	159.08	132.04
Slc44a2	318.49	3.64E-02	0.777	1.25	58.85	46.94
Nova1	71.73	3.65E-02	0.777	1.47	14.06	9.59
Mpeg1	1977.30	3.67E-02	0.777	1.20	358.50	298.14
Dock10	674.54	3.70E-02	0.777	1.22	123.49	101.01
Sf1	396.15	3.71E-02	0.777	1.25	72.77	58.24
Ptk2b	224.37	3.73E-02	0.777	1.30	41.61	32.05
Polr1c	68.05	3.74E-02	0.777	-1.49	8.92	13.30
Foxred1	63.21	3.74E-02	0.777	-1.47	8.44	12.44
Kif5a	1392.68	3.75E-02	0.777	1.30	255.10	196.60
Dhx9	259.43	3.76E-02	0.777	1.28	48.36	37.92
AI662270	108.72	3.77E-02	0.777	-1.40	15.06	21.04
Ube2g2	105.51	3.77E-02	0.777	1.46	20.57	14.12
mt-Co1	4333.72	3.81E-02	0.777	1.20	782.92	652.41
Gm28661	8677.19	3.81E-02	0.777	1.20	1565.78	1304.25
Atp8a2	146.30	3.82E-02	0.777	1.34	27.95	20.87
Ddi2	201.25	3.82E-02	0.777	1.31	37.79	28.89
Hmga1	280.98	3.82E-02	0.777	1.28	52.46	41.04
Camkk2	389.51	3.85E-02	0.777	1.24	71.98	57.90

Wtip	67.12	3.85E-02	0.777	1.47	13.26	9.04
Cdc42se1	241.68	3.86E-02	0.777	1.31	45.55	34.72
Zfp277	126.66	3.86E-02	0.777	1.38	24.28	17.61
Rab1b	97.01	3.88E-02	0.777	1.47	18.81	12.81
Inpp5d	1229.96	3.88E-02	0.777	1.19	222.79	186.69
Scamp2	1883.42	3.88E-02	0.777	1.19	340.69	286.18
Usp21	254.96	3.89E-02	0.777	1.30	47.47	36.58
Ube2j2	117.96	3.92E-02	0.777	1.38	22.82	16.55
Rab32	177.19	3.94E-02	0.777	1.37	33.87	24.67
Vim	75.87	3.95E-02	0.777	-1.55	9.28	14.42
Kifap3	84.50	3.95E-02	0.777	1.43	16.36	11.40
Bicd2	321.70	3.97E-02	0.777	-1.25	47.75	59.50
Nme2	293.21	3.99E-02	0.777	-1.26	43.25	54.33
Dpp9	66.42	4.00E-02	0.777	1.48	13.11	8.88
Mrpl30	61.97	4.01E-02	0.777	1.46	12.24	8.39
Mlx	61.78	4.02E-02	0.777	1.46	12.22	8.35
Gmip	473.20	4.03E-02	0.777	1.24	87.25	70.14
Amdhd2	71.08	4.04E-02	0.777	-1.48	9.31	13.80
Gm8186	85.42	4.06E-02	0.777	1.45	16.43	11.35
Smc4	156.50	4.07E-02	0.777	1.35	29.81	22.04
Fbxl20	102.81	4.09E-02	0.777	1.38	19.87	14.36
Cep170	153.70	4.10E-02	0.777	-1.34	21.81	29.21
Rims1	74.81	4.12E-02	0.777	1.44	14.59	10.12
Gm49980	67.84	4.13E-02	0.777	1.48	13.12	8.88
Pf4	103.48	4.16E-02	0.777	-1.40	14.22	19.84
Ipo4	68.14	4.16E-02	0.777	1.48	13.39	9.05
Smad3	266.51	4.16E-02	0.777	-1.29	38.44	49.74
Kif1a	101.28	4.17E-02	0.777	1.41	19.56	13.89
Med12	82.15	4.17E-02	0.777	1.48	16.13	10.92
Sfi1	228.73	4.18E-02	0.777	-1.31	32.70	42.78
Zbtb20	432.39	4.18E-02	0.777	1.23	79.38	64.74
Rnf166	233.35	4.19E-02	0.777	1.29	43.79	34.06
Sqor	177.97	4.20E-02	0.777	1.32	33.48	25.31
Dhrs1	128.10	4.21E-02	0.777	-1.37	17.63	24.21
Irf2bp1	118.03	4.21E-02	0.777	1.40	22.43	15.99
Taok3	504.48	4.21E-02	0.777	1.22	92.52	75.60
Rhoc	79.84	4.22E-02	0.777	-1.45	10.64	15.40
Uqcr10	166.39	4.23E-02	0.777	1.33	31.36	23.60
Skiv2l	135.15	4.23E-02	0.777	1.40	26.08	18.57
Sh3bp5	196.35	4.23E-02	0.777	1.31	37.01	28.29
H1f2	117.37	4.25E-02	0.777	1.37	22.49	16.44
Wdr83os	98.78	4.25E-02	0.777	1.38	19.07	13.79
Ang	290.09	4.28E-02	0.777	1.27	53.94	42.32
Nfatc3	262.38	4.28E-02	0.777	1.28	49.02	38.23
Tlr6	104.00	4.31E-02	0.777	1.42	20.00	14.12
mt-Co3	10955.70	4.32E-02	0.777	1.18	1968.09	1669.09
Chmp4b	308.19	4.35E-02	0.777	1.24	56.93	45.82
Atad1	184.79	4.36E-02	0.777	1.32	34.60	26.28
Ldb1	310.05	4.37E-02	0.777	1.27	57.64	45.40
Hadha	229.04	4.38E-02	0.777	1.28	42.87	33.37
Cbr2	68.84	4.42E-02	0.777	-1.49	8.95	13.31
Ngp	554.44	4.42E-02	0.777	2.37	70.69	29.88
Map3k1	140.74	4.42E-02	0.777	1.36	26.85	19.74
Gde1	148.12	4.45E-02	0.777	1.36	27.93	20.53
Ankrd12	651.19	4.45E-02	0.777	1.22	119.05	97.68
B4galt1	847.90	4.46E-02	0.777	1.21	153.71	126.71
Mrpl22	71.40	4.47E-02	0.777	1.45	13.96	9.62
Hbp1	271.27	4.48E-02	0.777	1.27	50.17	39.61
Gtf3c1	94.05	4.49E-02	0.777	1.39	18.24	13.12
Bmf	202.03	4.49E-02	0.777	1.29	37.89	29.31
Psap	10577.43	4.50E-02	0.777	1.17	1897.94	1623.48
Tango2	160.29	4.50E-02	0.777	1.33	30.29	22.70
Rab27a	96.23	4.54E-02	0.777	1.43	18.67	13.10
Eno3	71.52	4.55E-02	0.777	-1.48	9.46	13.98

Itch	199.53	4.55E-02	0.777	1.28	37.35	29.08
Gng12	92.62	4.56E-02	0.777	1.41	17.91	12.73
Ctdsp1	179.20	4.56E-02	0.777	1.33	33.82	25.42
Samhd1	450.36	4.58E-02	0.777	1.23	82.78	67.17
Uqcrc2	293.28	4.59E-02	0.777	1.25	54.39	43.45
Ccng1	122.12	4.60E-02	0.777	-1.36	17.17	23.40
Sox4	1302.62	4.61E-02	0.777	-1.18	199.06	235.13
Extl3	489.75	4.63E-02	0.777	1.22	89.30	73.28
Ikbkg	68.88	4.63E-02	0.777	1.48	13.46	9.09
Stimate	63.44	4.64E-02	0.777	1.50	12.39	8.28
Mtor	65.45	4.66E-02	0.777	1.46	12.76	8.74
Srsf5	1402.88	4.67E-02	0.777	1.18	253.20	213.94
Dgat1	85.83	4.68E-02	0.777	1.44	16.57	11.51
Srm	94.83	4.70E-02	0.777	1.39	18.23	13.12
Mafb	2241.96	4.72E-02	0.777	1.18	403.58	342.09
Osbpl7	63.21	4.72E-02	0.777	1.45	12.43	8.55
Pgm2l1	77.36	4.72E-02	0.777	-1.42	10.56	15.03
Gm8226	65.46	4.74E-02	0.777	1.43	12.81	8.93
Fance	146.66	4.76E-02	0.777	1.34	27.77	20.79
Mfap1b	261.28	4.76E-02	0.777	1.26	48.20	38.34
Abr	207.94	4.79E-02	0.777	1.28	38.80	30.30
Rnf149	556.97	4.80E-02	0.777	1.21	101.53	83.67
Rgs7bp	135.84	4.80E-02	0.777	-1.38	18.88	26.00
Polr2l	189.16	4.81E-02	0.777	-1.50	22.13	33.26
Il16	513.44	4.82E-02	0.777	1.21	93.70	77.43
Gm21596	103.83	4.82E-02	0.777	1.37	19.95	14.52
Ip6k2	65.50	4.87E-02	0.782	-1.46	8.77	12.79
Gm13166	280.50	4.87E-02	0.782	-1.25	41.48	51.90
Kif21b	568.15	4.90E-02	0.784	1.21	103.63	85.53
Nt5c2	85.29	4.91E-02	0.784	-1.44	11.51	16.58
Blvrb	120.22	4.96E-02	0.784	1.37	22.94	16.74
Gm28437	11634.36	4.97E-02	0.784	1.17	2084.88	1778.24
Lyz2	1079.93	4.98E-02	0.784	1.29	195.99	152.38
